# A Deep Dive into the Complex Chemical Mixture and Toxicity of Tire Wear Particle Leachate in Fathead Minnow

**DOI:** 10.1002/etc.5140

**Published:** 2021-08-02

**Authors:** Leah Chibwe, Joanne L. Parrott, Kallie Shires, Hufsa Khan, Stacey Clarence, Christine Lavalle, Cheryl Sullivan, Anna M. O'Brien, Amila O. De Silva, Derek C.G. Muir, Chelsea M. Rochman

**Affiliations:** ^1^ Department of Ecology and Evolutionary Biology University of Toronto, Toronto Ontario Canada; ^2^ Aquatic Contaminants Research Division Environment & Climate Change Canada, Burlington Ontario Canada

**Keywords:** Tire wear particles, Tire toxicity, Fathead minnow, Complex chemical mixtures

## Abstract

The ecological impact of tire wear particles in aquatic ecosystems is a growing environmental concern. We combined toxicity testing, using fathead minnow *(Pimephales promelas*) embryos, with nontarget high‐resolution liquid chromatography Orbitrap mass spectrometry to characterize the toxicity and chemical mixture of organic chemicals associated with tire particle leachates. We assessed: 1) exposure to tire particle leachates after leaching for 1‐, 3‐, and 10‐d; and 2) the effect of the presence and absence of small tire particulates in the leachates. We observed a decrease in embryonic heart rates, hatching success, and lengths, as well as an increase in the number of embryos with severe deformities and diminished eye and body pigmentation, after exposure to the leachates. Overall, there was a pattern whereby we observed more toxicity in the 10‐d leachates, and greater toxicity in unfiltered leachates. Redundancy analysis showed that several benzothiazoles and aryl‐amines were correlated with the toxic effects observed in the embryos. These included benzothiazole, 2‐aminobenzothiazole, 2‐mercaptobenzothiazole, N,N′‐diphenylguanidine, and N,N′‐diphenylurea. However, many other chemicals characterized as unknowns are likely to also play a key role in the adverse effects observed. Our study provides insight into the types of chemicals likely to be important toxicological drivers in tire leachates, and improves our understanding of the ecotoxicological impacts of tire wear particles. *Environ Toxicol Chem* 2022;41:1144–1153. © 2021 The Authors. *Environmental Toxicology and Chemistry* published by Wiley Periodicals LLC on behalf of SETAC.

## Graphical abstract

Redundancy analysis (RDA) shows positive correlations between chemicals benzothiazoles and aryl‐amines in tire leachates, and increased hatch severity and lack of pigmentation in fathead minnow embryos. OHBT = hydroxybenzothiazole; PBI = 3‐phenyl‐1,3‐benzothiazol‐2‐imine; NHBT = 2‐aminobenzothiazole; MeSBT = 2‐(methylthio)benzothiazole; MBT = 2‐mercaptobenzothiazole; DPG = N,N′‐diphenylguanidine; CPU = cyclohexyl‐3‐phenylurea; DHU = N,N′‐dicyclohexylurea; DPU = N,N′‐diphenylurea; DHPA = 4,5‐diphenyl‐1H‐pyrazole‐3‐amine; DHA = dicyclohexylamine; HM = hexa(methoxymethyl)melamine; ABZ = 3‐aminobenzamide; BT = benzothiazole; ACT = acetoacetanilide; TMQ = 1,2‐Dihydro‐2,2,4‐trimethylquinoline; ACR = acridene.

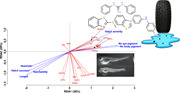

## INTRODUCTION

There is growing evidence that tire and road wear particles (TRWPs) constitute a substantial proportion of microplastics in the environment (Kole et al. 2017; Sommer et al. 2018; Järlskog et al. 2020). These particles are released from the frictional abrasion that occurs between tire tread and road surfaces. Thousands of tonnes of global TRWP emissions/yr have been estimated (Baensch‐Baltruschat et al. 2020), with model‐based studies suggesting they can account for up to 40% of microplastics exported to seas and rivers (Siegfried et al. 2017). Black rubber‐like fragments resembling TRWPs accounted for 22 and 50% of microplastics detected in stormwaters in the Greater Toronto and San Francisco Bay area, respectively (Sutton et al. 2019; Grbić et al. 2020). These particles have also been reported in sediments and in the digestive tracts of several fish species, including mullet (*Mugil cephalus*) and seatrout (*Cynoscion nebulosus*; Leads and Weinstein 2019; Sutton et al. 2019; Parker et al. 2020).

TRWPs enter aquatic environments through wet and dry deposition, such as during rain, wind, and storm events, and have led to concerns regarding the exposure of organisms to toxic chemicals. Various adverse effects have been reported when organisms were exposed to tire particles (i.e., particles manually generated from tire tread for testing purposes as an approximate to TRWPs; however, such tire particles do not represent aging and other environmental processes that bona fide TRWPs undergo, nor are they a composite of tire and road surface/asphalt particles) and to tire particle–derived leachates (Halle et al. 2020). These include reduction in the reproductive output and survival rates in the freshwater amphipod (*Hyalella azteca)* and soil worms (*Enchytraeus crypticus*; Khan et al. 2019; Ding et al. 2020), and oxidative stress in rainbow trout *(Oncorhynchus mykiss*; Stephensen et al. 2005). Some studies report no adverse effects and suggest that only leachates prepared from tire particles under harsh conditions (e.g., high temperatures and/or aggressive mechanical shaking) are toxic to aquatic taxa (Marwood et al. 2011; Panko et al. 2013). However, leachates prepared from tire particles at temperatures as low as 4 °C under nonaggressive conditions negatively affected fathead minnow (*Pimephales promelas*) embryos (Kolomijeca et al. 2020).

The analysis of the cocktail of additives in tires and tire‐derived leachates is challenging because the chemical formulations in tires are proprietary and complex. Tires generally contain approximately 60% of a styrene butadiene rubber polymer, fillers such as carbon black and silica, and many inorganic and organic chemicals added to aid in manufacture and promote certain properties, including elasticity, durability, and resistance to degradation (Babbit 1978; Hibbs 1990). Although knowledge about the organic composition of tires is still emerging, current strategies such as liquid chromatography high‐resolution mass spectrometry (LC–HRMS) and multidimensional gas chromatography mass spectrometry (2DGC–MS) have identified chemicals including cyclic amines and hexa(methoxymethyl)melamine in tire particle leachates (Peter et al. 2018; Capolupo et al. 2020; Halsband et al. 2020; Seiwert et al. 2020). A recent study demonstrated that tire wear particle leachates had a similar chemical signature to road runoff samples that induced mortality in coho salmon (*Oncorhynchus kisutch*; Peter et al. 2018). Other chemicals, such as polycyclic aromatic hydrocarbons (PAHs), benzothiazoles, and chlorinated paraffins have also been detected in tire rubber materials (Engler 2012; Llompart et al. 2013; Li et al. 2016; Brandsma et al. 2019).

The present study builds on prior work that reported severe deformities and a lack of pigmentation in fathead minnow embryos exposed to Bridgestone and Michelin tire particle leachates with the latter tire type yielding greater effects (Kolomijeca et al. 2020). Although zinc and PAHs were qualitatively determined in the samples, the leachates are likely to contain a more complex mixture of chemicals. The objectives of our study were to identify and assess the potency of organic chemicals in the same brand of Michelin tire particle leachates by combining toxicity analysis, using fathead minnow embryos, and nontarget HRMS. Specifically, we considered 2 factors: 1) exposure to tire particle leachates after leaching for 1‐, 3‐, and 10‐d to assess whether the toxicity increases or decreases over time; and 2) whether the presence of tire particulates in leachate samples enhances toxic effects. Our study provides insight into the chemical classes of interest relevant to the toxicity of tires, which is fundamental for determining priority organic substances in ecosystems receiving road runoff and informing solutions to tire particle toxicity.

## MATERIALS AND METHODS

### Chemicals and materials

Reference chemical standards (>98%) were purchased from Sigma Aldrich and Toronto Research Chemicals and included phenols, amines, and benzotriazoles (Supplemental Data, Table S1). The organic solvents methanol (MeOH) and acetonitrile (ACN) were HPLC grade and were purchased from EMD Chemicals. The HPLC and Optima grade water used for solid phase extraction (SPE) and instrumental analyses, respectively, were purchased from Fisher Chemicals.

### Tire particles generation and leachate preparation

Tire particles were generated as described previously (Kolomijeca et al. 2020; O'Brien et al. 2021). A new Michelin tire (Energy‐Saving, all season, sidewall markings, 205/60R16 91 V; Amazon 2018) was used because it elicited a greater toxic response in fathead minnow embryos compared with Bridgestone (Kolomijeca et al. 2020). Briefly, pieces of tire tread were cut using a pair of scissors and scalpel into approximately 5‐ × 5‐mm pieces. These pieces were finally ground using a high‐powered food processor and liquid nitrogen. Tire particle sizes ranged between 1.7 µm and 1.7 mm, with surface areas between 0.002 μm^2^ and 0.9 mm^2^ (Supplemental Data, Figure S1; O'Brien et al. 2021). Leachate samples were prepared by incubating 10 g/L of tire particles without agitation in 750‐mL amber capped glass bottles of control laboratory water (Burlington [ON, Canada] city water that was carbon‐filtered, ultraviolet (UV) light–sterilized in the laboratory, and sourced from Lake Ontario [ON, Canada]) in an environmental chamber at 34 °C. The exposure concentration was chosen because it has been observed to cause a negative effect in fathead minnow embryos (Wik and Dave 2005; Kolomijeca et al. 2020). Leaching occurred at 34 °C because prior research demonstrated toxicity when tire particles were subjected to leaching at this temperature (Kolomijeca et al. 2020). This also represents high recorded summer temperatures in the Greater Toronto Area (ON, Canada).

### Experimental design

The exposure study used a full‐factorial 2‐factor analysis of variance (ANOVA) design, with the following as factors: days of leaching (0 d = no tire; 1, 3, and 10 d) and whether the leachate was filtered or unfiltered (Supplemental Data, Figure S2A). We specifically considered 1 and 3 d of leaching to determine how toxicity compares with the 10‐d leachates (Kolomijeca et al. 2020). We wanted to investigate how the toxicity varies over time and determine what kinds of chemicals were driving the differences in toxic responses between days. Experiments were staggered so that leachate mixtures were simultaneously ready for the start of the subsequent 5‐d embryo toxicity exposures (i.e., leaching for the 10‐d samples started 9 d prior to the 1‐d, etc.). Half of the leachate was then passed through a 1‐mm mesh stainless steel sieve (“unfiltered”) and the other through a 0.45‐µm Whatman glass fiber filter (Sigma Aldrich) under light vacuum (“filtered”). Laboratory water was processed similarly, representing the “no tire” (0 d) filtered and unfiltered control, for a total of 8 treatments (Supplemental Data, Figure S2A). We included 3 replicates/treatment (*n* = 3), each conducted over 3 consecutive days (see below and Supplemental Data, Figure S2).

### Fathead minnow exposures

All embryo experiments were performed under Animal Use Standard Operating Protocol GWACC‐119, approved by the Animal Care Committee (operated under the approval of the Canadian Council of Animal Care [2010], at Environment and Climate Change Canada's Aquatic Life Research Facility [Burlington, ON, Canada]). The embryo exposures were performed in environmental chambers at controlled temperature and light (25 °C, 16:8‐h light: dark). Embryos were exposed using daily static renewal methods in 24‐well polystyrene cell culture plates (Falcon; Becton Dickinson; Marentette et al. 2015). The water conductivity ranged from 345 to 365 µS/cm, pH from 8.03 to 8.12, dissolved oxygen from 8.00 to 8.30 mg/L, temperature from 23.9 to 24.2 °C, and free or un‐ionized ammonia from 0.000 to 0.100 mg/L in the leachate solutions (Supplemental Data, Table S2); given the subtle differences and comparability to controls (water conductivity: 344–387 µS/cm; pH: 7.96–8.15; dissolved oxygen: 7.85–8.39 mg/L; temperature: 23.8–24.1 °C; and free ammonia 0.000–0.05 mg/L), we did not expect the water quality parameters to have a significant impact on embryos (Organisation for Economic Co‐operation and Development 1994).

Fathead minnow embryo exposure experiments began 1 d after the tire particle leachates were prepared. Leachates were tested without dilution at 100% concentration (10 g/L). Individual exposures of newly fertilized fathead minnow embryos (Aquatox Laboratories) were in 2‐mL wells. Toxicity exposure experiments were conducted over 3 consecutive days (running one replicate for each treatment/d), with eggs from ≥4 breeding groups used to begin each replicate (Supplemental Data, Figure S2B; note: leachate mixture solutions were not freshly prepared for each consecutive toxicity replicate). Exposure solutions were renewed daily, and leachate mixture solutions were stored at 4 °C in the dark throughout the experiments. To determine whether there were differences among days, 2 24‐well plates of control water (Burlington city water sourced from Lake Ontario and dechlorinated and UV‐sterilized in the laboratory) were used as interday controls. Each exposure plate, which was one replicate/treatment, contained 24 embryos (one/2‐mL well). The same water was also used for interplate controls. On each plate, 20 of the embryos were exposed to an experimental treatment, and 4 embryos were exposed to control water (Supplemental Data, Figure S2B). At 2 d post fertilization (dpf), 5 embryos/plate were video‐recorded to count heart rates. At 4 to 5 dpf, embryos began to hatch, and time of hatch was noted for each. Hatched embryos were assessed for deformities, hatch success (the percentage of embryos that hatched into viable fry), hatchability (the percentage of embryos that hatched), and length (measured on a dissecting microscope). The fathead minnow fry were euthanized by crushing (Canadian Council on Animal Care 2010). Eleutheroembryos (still feeding via yolk sac, before the onset of exogenous feeding; Belanger et al. 2010) are treated as a tissue culture under animal care guidelines as per the European Commission (2010) Directive on the protection of animals used for scientific purposes. Several deformities were monitored in the embryos and were based on a precedent of deformities in this species to other toxicants. These were: yolk, cardiac, and eye edema, kyphosis, lordosis, scoliosis (graded as severe, moderate, or mild), helix coil (severe), truncated body, necrosis, hemorrhage (severe or mild), and other deformities such as tube heart, bubbles under skin, small face, fused jaw, pin eyes, ruffled finfold, and bent tail. Embryos were also assessed for eye and body pigment (normal, some loss, and no pigment). Detailed information about the protocols and exposure design are provided in the Supplemental Data.

### Leachate extraction

Approximately 8 mL of the filtered tire particle leachates and no tire controls were used for chemical analyses and extracted within 3 d of prepared leachates. Prior to extraction, samples were spiked with isotopically ^13^C‐labeled surrogates. Samples were extracted in triplicate using reverse‐phase OASIS HLB (Waters) SPE for the isolation of acidic, basic, and neutral organic substances. Elution was conducted using 6 mL × 2 of acetone, and solvent exchanged to acetonitrile 3 times for a final volume of 1 mL. Prior to analysis, extracts were spiked with ^13^C‐labeled internal standards to evaluate matrix effects on instrument response. Detailed methods are provided in the Supplemental Data.

### Instrumental and data analysis

Chemical analysis was conducted on a Vanquish HPLC device coupled to a Q Exactive Focus Orbitrap MS (Thermo Fisher Scientific). An Acquity UPLC BEH C_18_ column (2.1 × 50 mm, 1.7 μm particle size (Waters) was used for chromatographic separation with mobile phases water (A) and methanol (B). The Full MS/data‐dependent acquisition‐ms^2^ discovery mode was used to acquire data in electrospray ionization (ESI) positive and negative modes, using a scan range of 100 to 1700 *m*/*z*, and fragmentation at 30 eV. Detailed parameters are provided in the Supplemental Data. The raw data were processed using Compound Discoverer 3.1 (Thermo Fisher Scientific), and included peak feature selection, peak alignment, adduct grouping, elemental formula assignment (<5 ppm mass tolerance), and mzCloud mass spectral library matching (>75%; Supplemental Data, Figure S3). The MZmine software (Pluskal et al. 2010) was used as a complementary tool to collaborate the Compound Discover data, with the mass detection, chromatogram builder, and peak deconvolution functions applied (Supplemental Data, Figure S4). The online MetFrag in silico prediction tool (Leibnitz Institute of Plant Biochemistry 2010) was used in addition, to aid in determining chemical class.

One hundred of the most abundant peaks with areas 5× the blank peak area detected in the filtered leachates were tentatively identified. The criteria used to convey the degree in confidence in identified peak features were as follows: level 1, spectral and retention time match with an authentic standard; level 2, spectral similarity MS/MS match with literature or the mzCloud database (>75%); level 3, characterization of functional group/class; and level 4, accurate mass and/or MS^2^ but no spectral or accurate mass matches.

We determined the concentrations for analytes with the authentic standards available at the time of the study (Supplemental Data, Table S1). We estimated concentrations for peaks with assigned structures (i.e., level 2), using 5‐ or 6‐point calibration curve and response factors from available standards based on elution time and physicochemical properties or structural similarity (further details are provided in the Supplemental Data). The peak area ratios of surrogate internal standards (spiked prior to extraction) to corresponding structurally related internal standards (spiked prior to analysis) in leachate samples indicated low recovery and/or evidence for matrix interferents or ion suppression (Supplemental Data, Figure S5). For these reasons, and given that the limited labeled standards used might not adequately represent the wide range of chemicals in the samples, the relative peak abundance (individual peak area/cumulative area of peaks in a given sample) was used for data analysis/interpretation. However, a brief discussion on estimated concentrations and comparisons with other studies is provided in the Supplemental Data, Table S3).

### Statistics and data analysis

Toxicity data were assessed for normality and homogeneity of variance using the Shapiro–Wilk and Levene's tests, respectively. Because 24‐well plate controls and in‐plate controls were used in toxicity exposure experiments to assess interday and intraplate variations, one‐way ANOVA analysis was used to compare these controls with the no tire filtered and unfiltered controls for the following endpoints: time to hatch, heart rate, hatchability (the percentage of embryos that hatched), hatching success (the percentage of embryos that hatched into viable fry), length at hatch, and proportion of embryos with severe deformities. There were no statistical differences between all controls for any endpoints (*p* > 0.05). Therefore, 2‐factor ANOVAs were used to assess the differences between each of the 8 treatment means relevant to time leached and filtration for each of the mentioned endpoints (α = 0.05, factor 1 = time [0, 1, 3, and 10 d of leaching]; factor 2 = filtered vs unfiltered). Tukey's honestly significant difference post hoc tests were used to identify significant differences between treatments groups for a significant factor or interaction (*p* < 0.05).

Principal component analysis (PCA) is an exploratory multidimensional data reduction technique that examines linear combinations of components for the most variation explained in a date set (Jolliffe and Cadima 2016). We used PCA to assess the chemical profile distributions between days of leaching in the filtered leachates. Redundancy analysis (RDA) is a multivariate technique that determines how much the variation in a set of response variables can be explained by the variation in explanatory variables (Van Den Wollenberg 1977). We used RDA to study how the variation in the toxic endpoints (response) between days of leaching correlated with the leached chemicals (explanatory). The RDA was limited to level 1 (authentic standard confirmation) and level 2 (mass spectral/literature match) peaks to explore relationships between chemicals with assigned structural features and toxic effects. Data were standardized (mean of 0, standard deviation of 1) prior to PCA and RDA. Analysis was conducted using R Studio (Ver 3.51). The ANOVAs, assumption checking, and post hoc tests were conducted using the *ggpubr, car, lsmeans*, and *multcompview* packages. The PCA and RDA were conducted using the *FactoMineR, factoextra*; and *vegan, faraway* packages, respectively.

### Quality control and assurance

All glassware used in the experiment was baked for at least 12 h at 450 °C and rinsed with acetone prior to use. Solvent MeOH blanks were analyzed every 5 to 6 samples to account for background contamination. For chemical analysis, instrument calibration was performed using the Pierce LTQ Velos ESI positive and negative ion calibration solutions (Thermo Scientific).

## RESULTS AND DISCUSSION

### Toxicity of filtered and unfiltered tire leachates to fathead minnow embryos

**Figure 1:**
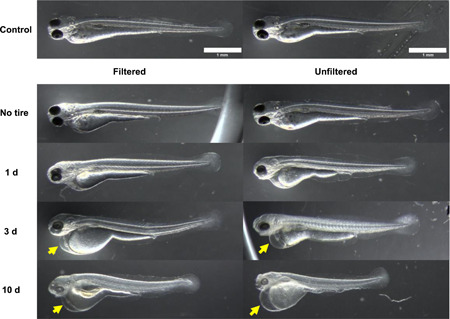
Effects observed in hatched fathead minnow fry after exposure to filtered and unfiltered tire particle samples, after 1, 3, and 10 d of leaching. The top 2 panels represent controls. Eye and body pigment levels as well as larval lengths decreased as leaching days increased. Severe cardiac edemas were observed in the 3‐ and 10‐d samples (yellow arrows) as well as blunted faces and malformed jaws.

Many deformities were present in embryos exposed to filtered and unfiltered tire particle leachates, including various edemas, as well as craniofacial defects, such as blunt faces or malformed jaws (Figure 1). A lack of eye and body pigmentation was also observed (Figure 1). The 3‐d unfiltered and 10‐d (filtered and unfiltered) leachates had the highest number of embryos with complete lack of eye and body pigment (Supplemental Data, Figure S6). Although the long‐term effects and implications of the lack of pigment in the fish remain to be understood, exposure to certain toxicants, including hydrazine and chlorinated phenols, has been linked with diminishing effects on pigmentation in fathead minnow (Henderson et al. 1981; Li et al. 2018). Several organic chemicals associated with pesticide or fungicide use, such as triclosan, 4‐chloroanaline, atrazine, and chlorpyrifos‐oxon, have also been tied to the lack of or abnormal skin and eye pigmentation effects in zebrafish (*Danio rerio*; McCollum et al. 2011).

Two‐factor analysis of variance (ANOVA; factor 1 = days leaching, factor 2 = filtered or unfiltered) was used to assess the differences between treatments for several endpoints: time to hatch, heart rate, length, hatchability (the percentage of embryos that hatched), hatch success (the percentage of embryos that hatched into viable fry), and proportion of embryos with severe deformities.

**Figure 2:**
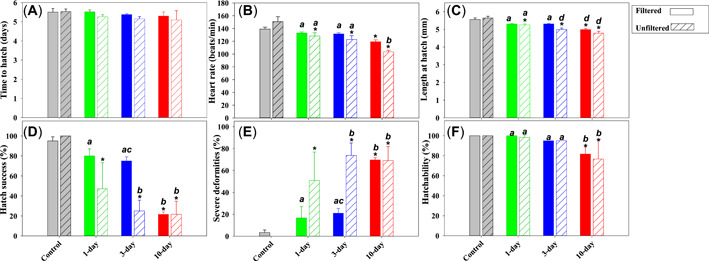
(**A–F**) Barplots of effects seen in fathead minnow embryos exposed to tire particle leachates. There were 3 replicates of each treatment. Hatchability is the percentage of embryos that hatched, and hatch success is the percentage of embryos that hatched into viable fry. Controls represent “no tire” control water exposures of dechlorinated charcoal filtered, ultraviolet light‐sterilized, treated Lake Ontario sourced laboratory control water. Asterisks (*) denote treatments that were significantly different from corresponding controls (i.e., filtered leachate vs filtered control, and unfiltered leachate vs unfiltered control; *p* < 0.05). Significant differences (*p* < 0.05) among leachate treatments are indicated by different letters (2‐way analysis of variance).

There were no significant differences among treatments for time to hatch, although there was a general decreasing trend with increasing days of leaching (*p* > 0.05; Figure 2A). There were significant interactions between days of leaching and filter for embryonic heart rate, length at hatch, hatch success rate, and the number of embryos with severe deformities (*p* < 0.05; Figure 2B–E and Supplemental Data, Table S4). Generally, for the unfiltered leachates, embryos exposed to the 1‐, 3‐, and 10‐d samples had lower heart rates, shorter lengths, lower hatch success rates, and more severe deformities than the corresponding unfiltered controls (*p* < 0.05; Figure 2B–E). For the filtered leachates, embryos exposed to the 10‐d samples had lower heart rates, shorter lengths, lower hatch success rates, and more severe deformities than the correponding filtered controls (*p* < 0.05; Figure 2B–E).

A trend showing a decrease in heart rates, lengths, and hatching success, and an increase in severe deformities, with increased days of leaching was evident (Figure 2). The 10‐d unfiltered leachate group had lower heart rates than the 1‐ and 3‐d unfiltered leachate group, and the 3‐ and 10‐d unfiltered leachate groups had shorter lengths than the 1‐d unfiltered leachate group (*p* < 0.01; Figure 2B and C). Moreover, the 10‐d filtered leachate group had significantly shorter lengths, lower hatch success rates, and more severe deformities than the 1‐ and 3‐d filtered leachate treatments (*p* < 0.05; Figure 2B–E). Similar to hatch success rates, hatchability rates decreased with increased days of leaching (Figure 2F). There was a difference between days of leaching *(p* = 0.004), with embryos exposed to 10‐d leachates having significantly lower hatchability rates than all other treatments, including controls (*p* < 0.05; Figure 2F).

Another hypothesis of our study was that the presence of small tire particles in conjunction with leachate would have an enhanced effect compared with leachate alone. Indeed, differences between the filtered and unfiltered groups for the 3‐d leachates were observed. Embryos in unfiltered leachates had shorter lengths, lower hatching success, and greater deformities than the embryos in filtered leachates (*p* < 0.009; Figure 2). This is consistent with recent research showing higher chemical concentrations and greater toxicity in freshwater *H. azteca* from tire wear particle suspensions than from leachates (Halle et al. 2021). It is beyond the scope of the present study to identify the specific mechanism of particles enhancing toxicity, but several possibilities warrant further exploration. The presence of particles could play a role in the chemical uptake and bioavailability of toxicants to organisms (LaPlaca and van den Hurk 2019). In addition, it is likely that there are differences in the chemical composition between the filtered and unfiltered leachates (Halle et al. 2021). It is also important to note that there was greater variation between replicates from exposure to the unfiltered leachates than the variation in replicates in the filtered exposures. This could reflect more variability in the particle sizes of the unlfiltered leachates compared with the filtered leachates (i.e., unfiltered leachates likely comprised a wide range of particle sizes ≤1 mm).

Overall, there was a pattern whereby we observed a greater extent of toxicity in the 10‐d leachate group, with lower heart rates, shorter lengths, lower hatch success and hatchability rates, and the most severe deformities. In other freshwater fish, exposure to stormwater containing chemicals from various sources, including tire wear, has been reported. Impaired growth and delayed hatching was observed in zebrafish (*D. rerio*) embryos (McIntyre et al. 2014), lateral line defects were reported in larval zebrafish and coho salmon embryos (Young et al. 2018), and abnormal larval or fish development were noted in medaka (*Oryzia latipes*) and silverside (*Menidia beryllina*; Skinner et al. 1999). Although tire wear particles contain a composition of mostly uncharacterized chemicals, they are likely to play an important role in adverse stormwater‐induced effects.

### Characterization of filtered tire particle leachates

#### Characterization of chemicals in the filtered leachates

We used LC–HR Orbitrap MS to profile the organic chemicals in the filtered tire particle leachates. Many of the peaks detected in ESI negative mode did not elicit MS/MS fragmentation spectra under the instrumental conditions used, preventing mass spectral library matching or further feature characterization. In addition, with the exception of 5 peaks (2‐mercaptobenzothiazole [MBT], P4, 3‐phenyl‐1,3‐benzothiazol‐2‐imine [PBI], P15, and P22 in Supplemental Data, Table S5), there was no significant overlap between the positive and negative ionization modes. Because this was a preliminary screening study, nontarget analysis was conducted to quickly determine the types of chemicals present and driving the toxicity effects in the filtered leachate samples. As a result, many chemicals were tentatively identified by mass spectral library matching to the mzCloud library (>75%; Schymanski et al. 2014; Wang et al. 2018) or with the literature (level 2; Supplemental Data, Table S5).

The cyclic amines N,N′‐diphenylguanidine (DPG) > cyclohexyl‐3‐phenylurea (CPU) > N,N′‐dicyclohexylurea (DHU) and > N,N′‐diphenylurea (DPU) comprised some of the most abundant peaks in ESI positive mode (Supplemental Data, Figure S7A and B). Applied as an accelerator in the vulcanization of rubber and polymer materials (Brorström‐Lundén et al. 2009), DPG was also abundant in tire crumb rubber, road dust, and road run‐off (Peter et al. 2018; Seiwert et al. 2020). Peter et al. (2020) reported DPG and DHU at concentrations of 100 ± 16 and 18 ± 2 µg/g in tire wear; together with CPU, these compounds have been associated with the coho mortality signature in North America (Peter et al. 2018).

Benzothiazole and its derivatives were detected in dec reasing order: hydroxybenzothiazole (OHBT) > benzothiazole (BT) > PBI > 2‐aminobenzothiazole (NHBT) > 2‐(methylthio)benzothiazole (MeSBT) > 2‐MBT; Supplemental Data, Figure S7C). The Benzothiazoles are used as vulcanization accelerators in the production of rubber materials (Kreider et al. 2010). In 17 major tire brands from 8 countries, benzothiazole (21–175 µg/g) and OHBT (6.5–40 µg/g) were the most abundant of the 7 benzothiazoles considered (Zhang et al. 2018), and benzothiazole (2.3–155 µg/g) and MBT (0.1–2200 µg/g) were the most abundant in different types of tires (winter, summer, studded, nonstudded) assessed in Norway and Sweden (Avagyan et al. 2014; Asheim et al 2019). Although benzothiazoles have been suggested as markers for tire wear due to their prevalence in traffic‐impacted samples, including stormwaters near major highways and tunnel water (Zhang et al. 2018; Asheim et al. 2019; Johnsen and Bye 2019), they are high‐production chemicals with various other industrial applications, including as fungicides and antifreeze corrosion inhibitors (Reemtsma et al. 1995; Kloepfer et al. 2005).

There were several other abundant peaks in the leachate samples identified as level 4 unknowns (Supplemental Data, Figure S7 and Table S5). To date, a limited number of suspect or nontarget screening studies of tire extracts of leachate samples exist (Peter et al. 2018; Capolupo et al. 2020; Halsband et al. 2020; Seiwert et al. 2020). The lack of overlap in unidentified features with the other aforementioned studies could potentially indicate the differences in composition that exist between tire brands and types. Furthermore, the fact that many of the chemicals were not present in mass spectral libraries highlights the need for shared high‐resolution MS spectral libraries in the risk assessment of tire wear.

**Figure 3:**
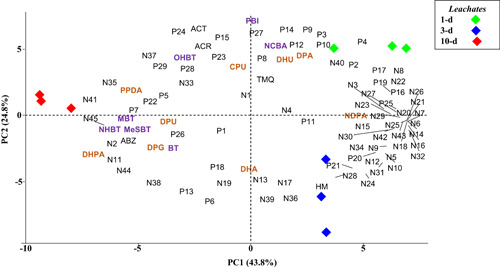
Principal component (PC) analysis of leachate replicates and variables (relative peak areas and effects). “P” and “N” represent unknowns (level 4) in positive and negative mode, respectively. Analytes in orange represent cyclic amines, and those in purple are the benzothiazole (BT) derivatives. Diamonds represent 1‐, 3‐, and 10‐d leachate replicates. Chemicals are plotted in coordinate space that represents the degree of loading on the axis. OHBT = hydroxybenzothiazole; PBI = 3‐phenyl‐1,3‐benzothiazol‐2‐imine; NHBT = 2‐aminobenzothiazole; MeSBT = 2‐(methylthio)benzothiazole; MBT = 2‐mercaptobenzothiazole; DPG = N,N′‐diphenylguanidine; CPU = cyclohexyl‐3‐phenylurea; DHU = N, N′‐dicyclohexylurea; DPU = N,N′‐diphenylurea; NCBA = N‐Cyclohexyl‐1,3‐benzothiazol‐2‐amine; NDPA = 4‐Nitrosodiphenylamine; DPA = diphenylamine; DHPA = 4,5‐diphenyl‐1H‐pyrazole‐3‐amine; DHA = dicyclohexylamine; HM = hexa(methoxymethyl)melamine.

The leaching behavior of chemicals assessed by their relative peak abundances in the samples varied. The relative abundances of some chemicals either remained the same or increased with increased days of leaching, as was observed for the amines and benzothiazoles (Supplemental Data, Figure S7A and B). Other chemicals decreased with increased days of leaching, whereas others peaked after 3 d of leaching, followed by a decline (Supplemental Data, Figure S7). The tire particle leachates likely encompass a complex mixture of chemicals with a wide range of physicochemical properties. These influence the interaction of the chemicals with the particles and water, and consequentially leaching trends. In addition, degradation or transformation reactions could result in some chemicals peaking after 3 d of leaching, or decreasing with increased days of leaching. To further explore the distributions for all the chemicals tentatively identified in the filtered leachate samples, we applied PCA. Using PCA, principal components 1 and 2 accounted for 43 and 25% of the total variance, respectively (Figure 3 and Supplemental Data, Table S6). There was generally separation between the 1‐, 3‐, and 10‐d leachate replicates, demonstrating the differences in chemical intensities between the days of leaching. The 10‐d leachates, which were generally the most toxic to fathead minnow embryos (Figures 1 and 2, and Supplemental Data, Figure S6), were correlated with peak areas of several chemicals, including several of the benzothiazoles: NHBT, MeSBT, MBT, and amines: DPG, DPU, 4,5‐diphenyl‐1H‐pyrazole‐3‐amine (DHPA) and N‐phenyl‐1,4‐benzene diamine (PPDA; Figure 3). Other chemicals associated with day 10 leachates included the unknowns N35, N41, N45, P5, P7, and P22 (Supplemental Data, Table S5), and might suggest similar leaching tendencies between these chemicals.

#### Relationships between levels 1 to 2 chemicals and toxicity in larval fish

**Figure 4:**
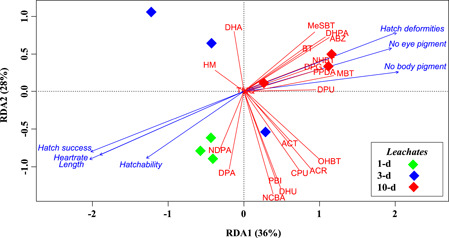
Projection of the samples onto axes representing the first axes of redundancy analysis (RDA) of the toxicity responses in fathead minnow embryos and level 1 and 2 chemicals in filtered leachate samples. Diamonds represent 1‐, 3‐, and 10‐d leachate replicates. Red arrows represent the chemicals (explanatory variables), and blue arrows represent the response variables (toxicity observations). Angles between explanatory and response vectors represent degree of correlation (angles >90° show no correlation). Vectors pointing in same direction also indicate positive correlation. ABZ = 3‐aminobenzamide; PPDA = N‐phenyl‐1,4‐benzene diamine; NDPA = 4‐nitrosodiphenylamine; ACR = acridene; ACT = acetoacetanilide. For other abbreviations, see Figure 3 legend.

An RDA was conducted to analyze the correlations between the toxicity responses and the 21 levels 1 and 2 chemicals in the filtered leachate replicates. We chose to focus on level 1 and 2 analytes because these chemicals were structurally characterized, and could provide more insight on how certain chemical classes correlated with the observed toxicological responses. The RDA produced an ordination in which the first 2 axes accounted for 64% of the variation in toxicity (Figure 4 and Supplemental Data, Table S7). The RDA ordination plot showed a strong positive association between the number of embryos that hatched with severe deformities and a lack of pigmentation (Figure 4). Furthermore, the chemicals MeSBT, DHPA, benzothiazole, NHBT, DPG, PPDA, MBT, and DPU were positively correlated with greater severe deformities and a lack of eye and body pigment, and negatively correlated with heart rate, hatching success, hatchability rate, and length (i.e., lower heart rates, lower hatching success, lower hatchability, and decreased lengths were associated with higher concentrations of these chemicals; Figure 4).

There were strong associations between several chemicals that belonged to the benzothiazole or aryl‐amine classes, and the adverse effects observed in the embryos from exposure to the tire particle leachates. In several leachates derived from tire rubber, samples containing benzothiazole accelerators and phenyl‐amine additives were also the most toxic to *Daphnia magna* (Wik et al. 2009). Benzothiazoles have been linked to numerous toxicological effects in humans and animals (Liao et al. 2018). For example, MBT, NHBT, and benzothiazole were cytotoxic and caused oxidative stress in 2 rainbow trout lines, and NHBT and benzothiazole showed evidence for genotoxicity (Zeng et al. 2016). Benzothiazole, MBT, and MeSBT bound to (and activated) the aryl hydrocarbon receptor in vitro (He et al. 2011), and MBT induced oxidative stress enzymes in livers of rainbow trout (Stephensen et al. 2005). The compound MBT has in addition been linked to skin sensitization in rodents and humans (Korhonen et al. 1983; Wang and Suskind 1988).

Comparatively limited toxicological data exist for aryl‐amines despite their prevalence in tire leachates and growing indications of their potential adverse effects in aquatic organisms (Peter et al. 2018). Exposure to sediment‐associated aryl‐amine antioxidants resulted in malformations, including severe cardiac, eye, and yolk edemas, in fathead minnow embryos and larvae (Prosser et al. 2017). More recently, an aryl‐amine transformation product, 2‐anilino‐5‐[(4‐methylpentan‐2‐yl)amino]cyclohexa‐2,5‐diene‐1,4‐dione, of the tire rubber antioxidant N‐(1,3‐dimethylbutyl)‐N′‐phenyl‐p‐phenylenediamine was identified in urban stormwater runoff and shown to induce acute toxicity in spawning coho salmon (*O. kisutch*; Tian et al. 2020). Altogether, these studies highlight that it is important to consider this class of chemicals, as well as their transformation products, in the exposure of aquatic organisms to tires.

## LIMITATIONS AND IMPORTANT CONSIDERATIONS

The results of our study suggest that several chemicals, including benzothiazole and its derivatives and aryl‐amines, likely contribute to the severe deformities and a lack of eye and body pigmentation in fathead minnow embryos. Many other chemicals with high abundances, and strong correlations with the toxic 10‐d leachates, could also be important toxicological drivers, but were detected as level 4 unknowns. Although metals were not analyzed, they could also play a key role in the toxicological responses observed, and their effects should be considered in combination with the organic chemicals identified. In addition, the tire particle leachate used in our exposure studies were generated without agitation. Thus the results of our study might not be directly comparable to tire particle leachate generated using a more aggressive procedure. It is likely that using a mechanical shaker or ultrasonication would enhance chemical partitioning from the tire particle into the leachate, and would likely render a more potent leachate. An important future goal in terms of the present study is to obtain authentic standards to confirm the level 2 chemicals, to conduct spike and recovery experiments to address extraction and instrumental bias as well as other factors including low recovery and matrix effects, and to subsequently determine concentrations. Overall, our study provides insight into the types of chemicals likely to be important toxicological drivers in tire leachates, and helps improve our understanding on the ecotoxicological impacts of tire wear.

## Supplemental Data

The Supplemental Data are available on the Wiley Online Library at https://doi.org/10.1002/etc.5140.

## Disclaimer

The authors declare no competing financial interest.

## Author Contributions Statement

The manuscript was written with contributions and feedback from all authors, and all authors have given consent to the final version of the manuscript. L. Chibwe planned and carried out the leachate preparation and chemical analyses experiments, and data analysis; J.L. Parrott planned and supervised the fathead minnow exposure experiments; K. Shires, S. Clarence, C. Lavalle, H. Khan, and C. Sullivan conducted the toxicity experiments and helped evaluate tox data; A.M. O'Brien helped prepare tire particles and plan leachate experiments; A.O. DeSilva and D.C.G. Muir helped plan the project, provided feedback on chemical analyses, and supervised; C.M. Rochman planned the project, provided funding, provided insights into statistical analyses and interpretation, and provided overall supervision of the work process.

## Supporting information

This article includes online‐only Supplemental Data.

Supporting information.

## Data Availability

Data, associated metadata, and calculation tools are available from the corresponding author (leahchibwe@gmail.com).

## References

[etc5140-bib-0001] Asheim J , Vike‐Jonas K , Gonzalez SV , Lierhagen S , Venkatraman V , Veivåg I‐LS , Snilsberg B , Flaten tire particle, Asimakopoulos AG. 2019. Benzotriazoles, benzothiazoles and trace elements in an urban road setting in Trondheim, Norway: Re‐visiting the chemical markers of traffic pollution. Sci Total Environ 649:703–711. 30176481 10.1016/j.scitotenv.2018.08.299

[etc5140-bib-0002] Avagyan R , Sadiktsis I , Bergvall C , Westerholm R . 2014. Tire tread wear particles in ambient air—A previously unknown source of human exposure to the biocide 2‐mercaptobenzothiazole. Environ Sci Pollut Res 21:11580–11586. 10.1007/s11356-014-3131-1 25028318

[etc5140-bib-0003] Babbit R , ed. 1978. *The Vanderbilt Rubber Handbook*. RT Vanderbilt, Norwalk, CT, USA.

[etc5140-bib-0004] Baensch‐Baltruschat B , Kocher B , Stock F , Reifferscheid G . 2020. Tyre and road wear particles (trwp)—A review of generation, properties, emissions, human health risk, ecotoxicity, and fate in the environment. Sci Total Environ 733:137823. 32422457 10.1016/j.scitotenv.2020.137823

[etc5140-bib-0005] Belanger SE , Balon EK , Rawlings JM . 2010. Saltatory ontogeny of fishes and sensitive early life stages for ecotoxicology tests. Aquat Toxicol 97:88–95. 20042243 10.1016/j.aquatox.2009.11.020

[etc5140-bib-0006] Brandsma SH , Brits M , Groenewoud QR , Van Velzen MJ , Leonards PE , De Boer J . 2019. Chlorinated paraffins in car tires recycled to rubber granulates and playground tiles. Environ Sci Technol 53:7595–7603. 31181880 10.1021/acs.est.9b01835 PMC6610544

[etc5140-bib-0007] Brorström‐Lundén E , Remberger M , Kaj L , Hansson K , Andersson H , Haglund P , Andersson R , Liljelind P , Grabic R. 2009. Screening of benzothiazoles, benzenediamines, dicyclohexylamine and benzotriazoles. ivl report b2023. Nordic Council of Ministers, Copenhagen, Denmark.

[etc5140-bib-0008] Capolupo M , Sørensen L , Jayasena KDR , Booth AM , Fabbri E . 2020. Chemical composition and ecotoxicity of plastic and car tire rubber leachates to aquatic organisms. Water Res 169:115270. 31731243 10.1016/j.watres.2019.115270

[etc5140-bib-0009] Canadian Council on Animal Care . 2010. CCAC guidelines on: Euthanasia of animals used in science. Ottawa, ON, Canada.

[etc5140-bib-0010] Ding J , Zhu D , Wang H‐T , Lassen SB , Chen Q‐L , Li G , Lv M , Zhu Y‐G . 2020. Dysbiosis in the gut microbiota of soil fauna explains the toxicity of tire tread particles. Environ Sci Technol 54:7450–7460. 32463658 10.1021/acs.est.0c00917

[etc5140-bib-0011] Engler RE . 2012. The complex interaction between marine debris and toxic chemicals in the ocean. Environ Sci Technol 46:12302–12315. 23088563 10.1021/es3027105

[etc5140-bib-0012] European Commission . 2010. Directive 2010/63/EU of the European Parliament and of the Council of 22 September 2010 on the protection of animals used for scientific purposes. Official J Eur Union L276:33–79.

[etc5140-bib-0013] Grbić J , Helm P , Athey S , Rochman CM . 2020. Microplastics entering northwestern Lake Ontario are diverse and linked to urban sources. Water Res 174:115623. 32088386 10.1016/j.watres.2020.115623

[etc5140-bib-0014] Halle LL , Palmqvist A , Kampmann K , Khan FR . 2020. Ecotoxicology of micronized tire rubber: Past. Sci Total Environ 706:135694. 31785900 10.1016/j.scitotenv.2019.135694

[etc5140-bib-0015] Halle LL , Palmqvist A , Kampmann K , Jensen A , Hansen T , Khan FR . 2021. Tire wear particle and leachate exposures from a pristine and road‐worn tire to *Hyalella azteca*: Comparison of chemical content and biological effects. Aquat Toxicol 232:105769. 33561741 10.1016/j.aquatox.2021.105769

[etc5140-bib-0016] Halsband C , Sørensen L , Booth AM , Herzke D . 2020. Car tire crumb rubber: Does leaching produce a toxic chemical cocktail in coastal marine systems? Front Environ Sci 8:125.

[etc5140-bib-0017] He G , Zhao B , Denison MS . 2011. Identification of benzothiazole derivatives and polycyclic aromatic hydrocarbons as aryl hydrocarbon receptor agonists present in tire extracts. Environ Toxicol Chem 30:1915–1925. 21590714 10.1002/etc.581 PMC3263332

[etc5140-bib-0018] Henderson V , Fisher J , D'Allessandris R . 1981. Toxic and teratogenic effects of hydrazine on fathead minnow (*Pimephales promelas*) embryos. Bull Environ Contam Toxicol 26:807–812. 7260452 10.1007/BF01622175

[etc5140-bib-0019] Hibbs J. 1990. Styrene‐butadiene rubbers. In Babbit T, ed, *The Vanderbilt Rubber Handbook*. RT Vanderbilt, Norwalk, CT, USA, pp 54–79.

[etc5140-bib-0020] Järlskog I , Strömvall A‐M , Magnusson K , Gustafsson M , Polukarova M , Galfi H , Aronsson M , Andersson‐Sköld Y . 2020. Occurrence of tire and bitumen wear microplastics on urban streets and in sweepsand and washwater. Sci Total Environ 729:138950. 32371211 10.1016/j.scitotenv.2020.138950

[etc5140-bib-0021] Johnsen JP , Bye NH. 2019. Assessment of tire wear emission in a road tunnel, using benzothiazoles as tracer in tunnel wash water. Norwegian University of Life Sciences, Ås, Norway.

[etc5140-bib-0022] Jolliffe IT , Cadima J . 2016. Principal component analysis: A review and recent developments. Philos Trans R Soc A Math Phys Eng Sci 374:20150202. 10.1098/rsta.2015.0202 PMC4792409 26953178

[etc5140-bib-0023] Khan FR , Halle LL , Palmqvist A . 2019. Acute and long‐term toxicity of micronized car tire wear particles to *Hyalella azteca* . Aquat Toxicol 213:105216. 31185428 10.1016/j.aquatox.2019.05.018

[etc5140-bib-0024] Kloepfer A , Jekel M , Reemtsma T . 2005. Occurrence, sources, and fate of benzothiazoles in municipal wastewater treatment plants. Environ Sci Technol 39:3792–3798. 15952387 10.1021/es048141e

[etc5140-bib-0025] Kole PJ , Löhr AJ , Van Belleghem FG , Ragas AM . 2017. Wear and tear of tyres: A stealthy source of microplastics in the environment. Int J Environ Res Public Health 14:1265. 10.3390/ijerph14101265 PMC5664766 29053641

[etc5140-bib-0026] Kolomijeca A , Parrott J , Khan H , Shires K , Clarence S , Sullivan C , Chibwe L , Sinton D , Rochman CM . 2020. Increased temperature and turbulence alter the effects of leachates from tire particles on fathead minnow (*Pimephales promelas*). Environ Sci Technol 54:1750–1759. 31904226 10.1021/acs.est.9b05994

[etc5140-bib-0027] Korhonen A , Hemminki K , Vainio H . 1983. Toxicity of rubber chemicals towards three‐day chicken embryos. Scand J Work Environ Health 9:115–119. 6648408 10.5271/sjweh.2435

[etc5140-bib-0028] Kreider ML , Panko JM , McAtee BL , Sweet LI , Finley BL . 2010. Physical and chemical characterization of tire‐related particles: Comparison of particles generated using different methodologies. Sci Total Environ 408:652–659. 19896165 10.1016/j.scitotenv.2009.10.016

[etc5140-bib-0029] LaPlaca SB , van den Hurk P . 2019. Toxicological effects of tire wear particles on mummichogs and fathead minnows. Ecotoxicology 29:524–534. 10.1007/s10646-020-02210-7 32342294

[etc5140-bib-0030] Leads RR , Weinstein JE . 2019. Occurrence of tire wear particles and other microplastics within the tributaries of the Charleston Harbor Estuary, South Carolina, USA. Mar Pollut Bull 145:569–582. 31590826 10.1016/j.marpolbul.2019.06.061

[etc5140-bib-0031] Leibnitz Institute of Plant Biochemistry . 2010. MetFrag. [cited 2020 May 14]. Available from: https://ipb-halle.github.io/MetFrag/

[etc5140-bib-0032] Li E , Bolser DG , Kroll KJ , Brockmeier EK , Falciani F , Denslow ND . 2018. Comparative toxicity of three phenolic compounds on the embryo of fathead minnow, *Pimephales promelas* . Aquat Toxicol 201:66–72. 29879596 10.1016/j.aquatox.2018.05.024

[etc5140-bib-0033] Li H‐X , Getzinger GJ , Ferguson PL , Orihuela B , Zhu M , Rittschof D . 2016. Effects of toxic leachate from commercial plastics on larval survival and settlement of the barnacle *Amphibalanus amphitrite* . Environ Sci Technol 50:924–931. 26667586 10.1021/acs.est.5b02781

[etc5140-bib-0034] Liao C , Kim U‐J , Kannan K . 2018. A review of environmental occurrence, fate, exposure, and toxicity of benzothiazoles. Environ Sci Technol 52:5007–5026. 29578695 10.1021/acs.est.7b05493

[etc5140-bib-0035] Llompart M , Sanchez‐Prado L , Lamas JP , Garcia‐Jares C , Roca E , Dagnac T . 2013. Hazardous organic chemicals in rubber recycled tire playgrounds and pavers. Chemosphere 90:423–431. 22921644 10.1016/j.chemosphere.2012.07.053

[etc5140-bib-0036] Marentette JR , Frank RA , Bartlett AJ , Gillis PL , Hewitt LM , Peru KM , Headley JV , Brunswick P , Shang D , Parrott JL . 2015. Toxicity of naphthenic acid fraction components extracted from fresh and aged oil sands process‐affected waters, and commercial naphthenic acid mixtures, to fathead minnow (*Pimephales promelas*) embryos. Aquat Toxicol 164:108–117. 25957715 10.1016/j.aquatox.2015.04.024

[etc5140-bib-0037] Marwood C , McAtee B , Kreider M , Ogle RS , Finley B , Sweet L , Panko J . 2011. Acute aquatic toxicity of tire and road wear particles to alga, daphnid, and fish. Ecotoxicology 20:2079–2089. 21789673 10.1007/s10646-011-0750-x PMC7270990

[etc5140-bib-0038] McCollum CW , Ducharme NA , Bondesson M , Gustafsson JA . 2011. Developmental toxicity screening in zebrafish. Birth Defects Res C 93:67–114. 10.1002/bdrc.20210 21671351

[etc5140-bib-0039] McIntyre J , Davis J , Incardona J , Stark JD , Anulacion B , Scholz N . 2014. Zebrafish and clean water technology: Assessing soil bioretention as a protective treatment for toxic urban runoff. Sci Total Environ 500:173–180. 25217993 10.1016/j.scitotenv.2014.08.066

[etc5140-bib-0040] O'Brien AM , Lins TF , Yang Y , Frederickson ME , Sinton D , Rochman CM. 2021. A common contaminant shifts impacts of climate change on a plant‐microbe mutualism: Effects of temperature, CO_2_ and leachate from tire wear particles. *bioRxiv*. [cited 2020 July 25]. Available from: 10.1101/2020.1105.1119.105098

[etc5140-bib-0041] Organisation for Economic Co‐operation and Developmentt . 1994. Fish, early‐life stage toxicity test. *OECD Guidelines for the Testing of Chemicals*, Section 2. Paris, France.

[etc5140-bib-0042] Panko JM , Kreider ML , McAtee BL , Marwood C . 2013. Chronic toxicity of tire and road wear particles to water‐and sediment‐dwelling organisms. Ecotoxicology 22:13–21. 23001428 10.1007/s10646-012-0998-9 PMC7329783

[etc5140-bib-0043] Parker BW , Beckingham BA , Ingram BC , Ballenger JC , Weinstein JE , Sancho G . 2020. Microplastic and tire wear particle occurrence in fishes from an urban estuary: Influence of feeding characteristics on exposure risk. Mar Pollut Bull 160:111539. 32781266 10.1016/j.marpolbul.2020.111539

[etc5140-bib-0047] Peter KT , Hou F , Tian Z , Wu C , Goehring M , Liu F , Kolodziej EP . 2020. More than a first flush: Urban creek storm hydrographs demonstrate broad contaminant pollutographs. Environ Sci Technol 54:6152–6165. 32302122 10.1021/acs.est.0c00872

[etc5140-bib-0045] Peter KT , Tian Z , Wu C , Lin P , White S , Du B , McIntyre JK , Scholz NL , Kolodziej EP . 2018. Using high‐resolution mass spectrometry to identify organic contaminants linked to urban stormwater mortality syndrome in coho salmon. Environ Sci Technol 52:10317–10327. 30192129 10.1021/acs.est.8b03287

[etc5140-bib-0044] Pluskal T , Castillo S , Villar‐Briones A , Orešič M . 2010. Mzmine 2: Modular framework for processing, visualizing, and analyzing mass spectrometry‐based molecular profile data. BMC Bioinform 11:1–11. 10.1186/1471-2105-11-395 PMC2918584 20650010

[etc5140-bib-0046] Prosser RS , Parrott JL , Galicia M , Shires K , Sullivan C , Toito J , Bartlett AJ , Milani D , Gillis PL , Balakrishnan VK . 2017. Toxicity of sediment‐associated substituted phenylamine antioxidants on the early life stages of *Pimephales promelas* and a characterization of effects on freshwater organisms. Environ Toxicol Chem 36:2730–2738. 28418159 10.1002/etc.3828

[etc5140-bib-0048] Reemtsma T , Fiehn O , Kalnowski G , Jekel M . 1995. Microbial transformations and biological effects of fungicide‐derived benzothiazoles determined in industrial wastewater. Environ Sci Technol 29:478–485. 22201395 10.1021/es00002a025

[etc5140-bib-0049] Schymanski EL , Jeon J , Gulde R , Fenner K , Ruff M , Singer HP , Hollender J. 2014. Identifying small molecules via high resolution mass spectrometry: Communicating confidence. ACS, Washington, DC. 10.1021/es5002105 24476540

[etc5140-bib-0050] Seiwert B , Klöckner P , Wagner S , Reemtsma T . 2020. Source‐related smart suspect screening in the aqueous environment: Search for tire‐derived persistent and mobile trace organic contaminants in surface waters. Anal Bioanal Chem 412:4909–4919. 32382968 10.1007/s00216-020-02653-1 PMC7334239

[etc5140-bib-0051] Siegfried M , Koelmans AA , Besseling E , Kroeze C . 2017. Export of microplastics from land to sea. A modelling approach. Water Res 127:249–257. 29059612 10.1016/j.watres.2017.10.011

[etc5140-bib-0052] Skinner L , De Peyster A , Schiff K . 1999. Developmental effects of urban storm water in medaka (*Oryzias latipes*) and inland silverside (*Menidia beryllina*). Arch Environ Contam Toxicol 37:227–235. 10398773 10.1007/s002449900509

[etc5140-bib-0053] Sommer F , Dietze V , Baum A , Sauer J , Gilge S , Maschowski C , Gieré R . 2018. Tire abrasion as a major source of microplastics in the environment. Aerosol Air Qual Res 18:2014–2028.

[etc5140-bib-0054] Stephensen E , Adolfsson‐Erici M , Hulander M , Parkkonen J , Förlin L . 2005. Rubber additives induce oxidative stress in rainbow trout. Aquat Toxicol 75:136–143. 16144723 10.1016/j.aquatox.2005.07.008

[etc5140-bib-0055] Sutton R , Franz A , Gilbreath A , Lin D , Miller L , Sedlak M , Wong A , Box C , Holleman R , Munno K. 2019. Understanding microplastic levels, pathways, and transport in the San Francisco bay region. SFEI‐ASC publication# 950. San Francisco Estuary Institute, San Francisoco, CA, USA.

[etc5140-bib-0056] Tian Z , Zhao H , Peter KT , Gonzalez M , Wetzel J , Wu C , Hu X , Prat J , Mudrock E , Hettinger R . 2020. A ubiquitous tire rubber–derived chemical induces acute mortality in coho salmon. Science 371:185–189. 33273063 10.1126/science.abd6951

[etc5140-bib-0057] Van Den Wollenberg AL . 1977. Redundancy analysis an alternative for canonical correlation analysis. Psychometrika 42:207–219.

[etc5140-bib-0058] Wang A , Gerona RR , Schwartz JM , Lin T , Sirota M , Morello‐Frosch R , Woodruff TJ . 2018. A suspect screening method for characterizing multiple chemical exposures among a demographically diverse population of pregnant women in San Francisco. Environ Health Perspect 126:077009. 30044231 10.1289/EHP2920 PMC6108847

[etc5140-bib-0059] Wang X , Suskind RR . 1988. Comparative studies of the sensitization potential of morpholine, 2‐mercaptobenzothiazole and 2 of their derivatives in guinea pigs. Contact Dermatitis 19:11–15. 3180764 10.1111/j.1600-0536.1988.tb02861.x

[etc5140-bib-0060] Wik A , Dave G . 2005. Environmental labeling of car tires—Toxicity to *Daphnia magna* can be used as a screening method. Chemosphere 58:645–651. 15620758 10.1016/j.chemosphere.2004.08.103

[etc5140-bib-0061] Wik A , Nilsson E , Källqvist T , Tobiesen A , Dave G . 2009. Toxicity assessment of sequential leachates of tire powder using a battery of toxicity tests and toxicity identification evaluations. Chemosphere 77:922–927. 19758678 10.1016/j.chemosphere.2009.08.034

[etc5140-bib-0062] Young A , Kochenkov V , McIntyre JK , Stark JD , Coffin AB . 2018. Urban stormwater runoff negatively impacts lateral line development in larval zebrafish and salmon embryos. Scientific reports 8:1–14. 29434264 10.1038/s41598-018-21209-z PMC5809384

[etc5140-bib-0063] Zeng F , Sherry JP , Bols NC . 2016. Evaluating the toxic potential of benzothiazoles with the rainbow trout cell lines, rtgill‐w1 and rtl‐w1. Chemosphere 155:308–318. 27131451 10.1016/j.chemosphere.2016.04.079

[etc5140-bib-0064] Zhang J , Zhang X , Wu L , Wang T , Zhao J , Zhang Y , Men Z , Mao H . 2018. Occurrence of benzothiazole and its derivates in tire wear, road dust, and roadside soil. Chemosphere 201:310–317. 29525659 10.1016/j.chemosphere.2018.03.007

