# Supplementary material for: A Deep Dive into the Complex Chemical Mixture and Toxicity of Tire Wear Particle Leachate in Fathead Minnow
Source: Environ Toxicol Chem. 2021 Aug 2;41(5):1144–53. doi: 10.1002/etc.5140 (PMC9291566; doi:10.1002/etc.5140)
Supplement: Supplementary file 1 — Supporting information. [file ETC-41-1144-s001.docx]

**A deep dive into the complex chemical mixture and toxicity of tire wear particle leachate in fathead minnow**

Leah Chibwe*^1^, Joanne L. Parrott^2^, Kallie Shires^2^, Hufsa Khan^2^, Stacey Clarence^2^, Christine Lavalle^2^, Cheryl Sullivan^2^_,_ Anna O’Brien,^1^ Amila O. De Silva^2^, Derek C. G. Muir^2^, Chelsea M. Rochman*^1^

^1^ University of Toronto, Department of Ecology and Evolutionary Biology, Toronto, ON, Canada

^2^Aquatic Contaminants Research Division, Environment & Climate Change Canada, Burlington, ON, Canada

Corresponding Authors:

*(C.M. R.): [chelsea.rochman@utoronto.ca](mailto:chelsea.rochman@utoronto.ca); ORCID: 0000-0002-2642-6574

*(L.C.): [leahchibwe@gmail.com](mailto:leahchibwe@gmail.com); ORCID: 0000-0003-4870-5192

*Corresponding Author note: *Address correspondence to [chelsea.rochman@utoronto.ca](mailto:chelsea.rochman@utoronto.ca)

**SUPPORTING INFORMATION**

**Figure S1.** Images of tires particles generated for tire leachate experiments.

**Figure S2.** Experimental two-way factorial design of tire particle leachate preparation for toxicity exposure experiments, and toxicity exposure set-up with additional controls to assess inter-day and intra-plate variations.

**Figure S3.** Compound Discoverer processing parameters for raw data.

**Figure S4**. mzMine data processing parameters for raw data.

**Figure S5.** The peak area ratios of surrogate (SS) (labelled standard spike prior to extraction) to internal standard (IS) (labelled standard spike prior to instrumental analysis in leachate samples, and the peak area ratios IS in leachate samples to calibration curves (same concentration spike) in positive and negative ESI modes.

**Figure S6**. Scoring of eye pigment at hatch and body pigment at hatch in fathead minnow fry exposed to “no tire” controls, 1-, 3- and 10-day filtered and unfiltered tire leachates.

**Figure S7**. Average relative area (individual/cumulative area in sample) for all peaks, cyclic amines, benzothiazoles identified in positive mode, and all peaks identified in negative mode.

**Table S1**. Retention time, calibration curve range, regression coefficients (R^2^), and average response factors determined from internal standards in positive mode.

**Table S2.** Data for water quality measured during the embryo exposures of fathead minnow tire leachates and control waters.

**Table S3**. Estimated concentrations of organic chemicals tire particle (TP) and TP leachates in present study compared to previous studies.

**Table S4.** Summary of two-way ANOVA results using factors days of leaching and filter for the toxicity endpoints studied.

**Table S5.** Chemical formula, exact mass of precursor ions and 100 most intense product ions observed of studied compounds.

**Table S6**. Principal Component Analysis loadings of all peak features in leachate samples.

**Table S7.** Eigenvalues and percentage of variance explained by the redundancy analysis.

**MATERIALS AND METHODS**

***Fathead Minnow Exposures***

Exposures of fathead minnow (*Pimephales promelas*) embryos were conducted at Environment and Climate Change Canada’s Aquatic Life Research Facility (Burlington, ON). Newly fertilized fathead minnow embryos were purchased from Aquatox Laboratories (Guelph, ON). The eggs used in testing had been fertilized < 18 h before the start of the exposure. Embryo exposure solutions were prepared by dispensing into 2 mL wells and plates were warmed to 25 ̊C, 1-2 h prior to experiments starting or 1-2 h prior to embryo exposure solution renewal. Individual embryo exposures were in 2 mL wells. Embryos were assessed and moved to new plates containing fresh exposure solutions daily. Any dead embryos were removed from plates. At 2 days post fertilization (dpf), embryos were swirled in plates to center them, and heartbeats were video recorded and counted independently by two individuals; if averages of counts disagreed by more than 3 beats, they were recounted by a 3^rd^ individual.

***Leachate Extraction***

Prior to extraction, filtered leachate samples were spiked with isotopically labeled surrogates: tris(2-butoxy) [^13^C_2_]ethylphosphate (M6TBEP), [^13^C_8_]-triphenyl phosphate (MTPP) and diphenyl-p-tolyl phosphate-d_7_) (p-tolyl-d_7_) for positive mode; and [^13^C_4_]-perfluorooctanesulfonate (MPFOS) and perfluoro-*n*-[1,2-^13^C]decanoic acid (MPFDA) for negative mode. Samples were extracted in triplicate using OASIS HLB (Waters Corporation, Mississauga) solid phase extraction (SPE). The SPE cartridges were conditioned with 6 mL acetone, followed by 6 mL water. The sample was loaded onto the SPE cartridge by gravity, followed by drying under a vacuum for 30 minutes. Elution was conducted using 6 mL x 2 of acetone. The combined extract was reduced to ~ 0.5 mL of acetone using a gentle stream of nitrogen and solvent exchanged to acetonitrile 3 times for a final volume of 1 mL. Prior to analysis, extracts were spiked with labelled internal standards to evaluate matrix effects on instrument response: triphenyl d_15_ phosphate (d_15_-TPP) and tris(2-butoxyethyl)phosphate-d_27_ (_d27-_TBEP) for positive mode; and perfluoro-n-[1,2,3,4,5,6-^13^C_6_]decanoic acid (M6PFDA) and sodium perfluoro-1-[^13^C_8_]octanesulfonate (M8PFOS) for negative mode.

***Instrumental Analysis and Data Analysis***

The LC method elution gradient was: 10% B at 1.5 min, 50% B at 4 min, 100% B at10 min, hold for 4 mins, 10% at 21 min, and a final hold for 1 min (total run time of 22 min). The injection volume was 5 μL with a flow rate of 0.2 mL/min at a 40 °C column temperature. The source conditions were: spray voltage: 5 kV (positive) and 4.5 kV (negative); auxiliary and capillary temperatures: 300 °C; sheath gas flow rate: 30 arbitrary units (a.u.); auxiliary gas: 5 a.u. The Full MS/dd-ms^2^ (data-dependent acquisition) discovery mode was used to acquire data, at resolution of 70,000 and 35,000 FWHM for MS1 and MS2, respectively, scan range of 100–1700 *m*/*z*, and an automatic gain control target of 3e6. Data dependent acquisition fragmentation was conducted at 30 eV. The adducts considered in Compound Discoverer 3.1 (Thermo Fisher Scientific, CA, USA) were ([M+H]^+^, [M+Na] ^+^, [M+NH_4_] ^+^ for positive mode and [M-H] ^-^ for negative mode) (Figure S3). The accurate masses and MS/MS (if available) were additionally cross-referenced against the literature and the Global Automotive Declarable Substance List (Johnsen and Bye 2019).

***Estimated concentrations***

Concentrations were determined using a five or six-point calibration curve of several chemical standards within the range 10-1000 pg/µL (Table S1). The response factors of each standard at each calibration level were determined using equation 1:

$$Response factor \mathbf{(RF)}=\frac{\left( \mathrm{Ax} \right). (Cis)}{\left( \mathrm{Ais} \right)(Cx)}$$

where Ax = area of authentic standard for that given calibration curve level, Cis = concentration of internal standard (same concentration spiked in samples prior to instrumental analysis, and across all calibration levels), Ais = area of internal standard and Cx = concentration of authentic standard for that given calibration level. An average RF across all calibration levels was then determined.

Concentrations in the samples were then estimated using equation (1) and the determined average RF, where Ax = area of target peak in sample, Cis = concentration of internal standard (same concentration spiked in samples and calibration curve prior to instrumental analysis), Ais = area of internal standard and **RF** = determined response factor from the calibration curve. For peaks without authentic standards (but with structure assignments, i.e., Level 2), the average RF factor of the two authentic standards nearest in retention time and structure was used (Table S1). For example for DPA (RT: 8.09 mins, log Kow = 3.5, solubility ~ 53 mg/L), the average of RFs of authentic standards TMQ (RT: 8.23 mins, log Kow = 3.3, solubility ~ 89 mg/L), and BhQ (RT: 7.89 mins, log Kow = 3.4, solubility ~ 53mg/L), was used, as they were the most similar in terms of physicochemical properties and RT. Solubility and Log Kow data was taken from NIH chemID plus website (<https://chem.nlm.nih.gov/chemidplus/>) or estimated using EPI Suite, if not available.

The estimated concentrations are presented in Table S3. The peak area ratios of surrogate internal standards (SS, spiked prior to extraction) to corresponding structurally related internal standards (ISTDs, spiked prior to analysis) in leachate samples were between 0.36 and 0.61, and 0.90 and 2.01 in positive and negative mode, respectively (Figure S5-A, C). The peak area ratios of ISTDs in samples to those in calibrations curves (same concentrations) were between 0.52 to 0.75, and 0.80 to 0.85 in positive and negative mode, respectively (Figure S5-B, D). The SS/ISTD ratios indicated low recovery and/or evidence for matrix interferents or ion suppression

Limited target analysis (and concentrations) of organics in tires or tire leachates exist for the many chemicals presented, with BT and several of its derivatives being the most commonly reported. Comparison to literature showed the estimated concentrations were generally significantly lower than those reported. There are likely many factors contributing to this such as the brand of tire and production variability. However, we also acknowledge that concentrations were estimated using a limited number of internal and surrogate standards, which might not be adequate for many of the chemicals assessed. Furthermore, we employed a very general extraction method to limit discrimination in chemical isolation. A consequence of this approach is that the extraction is not optimal for certain chemicals. The estimated concentrations do not take into account analyte loss during extraction and clean-up, instrument matrix effects, or low partitioning from the tire particles to the solvents used in extraction. Future directions include verifying the tentatively identified compounds, spike and recovery experiments to assess analyte loss and extraction bias, as well as investigating representative surrogate and internal standards, for a more reliable (semi) quantitative method.

**Figure S1.** Tires particles generated for tire leachate experiments at a scale of (A) 5 mm (B) 1 mm and (C) 5 µm. Tire particle sizes ranged between 1.7 µm to 1.7 mm and surface area between 0.002 μm^2^ to 0.9 mm^2^ (O’Brien et al. 2021).

**Figure S2.** (A) Experimental two-way factorial design of tire particle leachate preparation for toxicity exposure experiments (Factor 1 was the number of days of leaching and Factor 2 was whether the leachate was filtered or unfiltered for a total of 8 treatments), and (B) Toxicity exposure set-up with additional controls to assess inter-day and intra-plate variations. Leachate solutions were not freshly prepared for each toxicity replicate.

**Align Retention times**

MS tolerance: 5 ppm

Maximum shift: 0.5 min

Alignment Mode: Adaptive

**Detect Compounds**

Mass Tolerance: 5 ppm

Max. Element Count: 90 [13]C15 H120 Br20 Cl20 D40 F50 N15 Na O15 P10 S10

Min. Element Count: C

Min. Peak Intensity: 100000

S/N Threshold: 6

**Select spectra**

**Input files**

**Assign Compound Annotations**

MS tolerance: 5 ppm

Data Source 1: mzCloud Search

Data Source 1: Predicted Compositions

Data Source 1: MassList Searc

Data Source 1: ChemSpider Search

**Search Mass Lists**

MS tolerance: 5 ppm

RT Tolerance [min]: 0.5

**Group Compounds**

MS tolerance: 5 ppm

RT Tolerance [min]: 0.15

**Search mzCloud**

MS tolerance: 5 ppm

RT Tolerance [min]: 0.15

**Predict Compositions**

MS tolerance: 5 ppm

Max. Element Count: C90 H120 Br20 Cl20 F50 N15 O15 P10 S10

Max. H/C: 3

Max. # Candidates: 10

Max. RDBE: 40

Min. Element Count: C

Min H/C: 0.1

Min. RDBE: -40

Pattern Matching

Intensity Threshold: 1

Intensity Tolerance: 30

S/N Threshold: 3

Fragments Matching

Mass Tolerance: 5 ppm

S/N Threshold: 3

**Fill Gaps**

MS tolerance: 5 ppm

S/N Threshold: 5

**Pattern Scoring**

Intensity Tolerance: 30

Mass Tolerance: 3 ppm

Min. Spectral Fit [%]: 10

SN Threshold: 3

**Mark Background Compounds**

Max. Blank/Sample: 0

Max. Sample/Blank: 5

**Search ChemSpider**

MS tolerance: 5 ppm

**Figure S3.** Compound Discoverer processing parameters for raw data.

**Figure S4.** mzMine data processing parameters for raw data.

**Figure S5.** The peak area ratios of surrogate (SS) (labelled standard spike prior to extraction) to internal standard (IS) (labelled standard spike prior to instrumental analysis in leachate samples in (A) positive mode, and (C) negative mode. The peak area ratios IS in leachate samples to calibration curves (same concentration spike) in (B) positive mode and (D) negative mode. In (A) M6TBEP = M6TBEP/d27TBEP, MTTP = MTTP/d15TPP, and pTolyd7 = pTolyd7/d15TPP. In (C), MPFOS = MPFOS/M8PFOS and MPFDA = MPFDA/M6PFDA. Tris(2-butoxy)[^13^C_2_]ethylphosphate (M6TBEP); [^13^C_8_]-triphenyl phosphate (MTPP); diphenyl-p-tolyl phosphate-d_7_) (pTolyl-d_7_); triphenyl d_15_ phosphate (d_15_TPP); tris(2-butoxyethyl)phosphate-d_27_ (_d27_TBEP); [^13^C_4_]-perfluorooctanesulfonate (MPFOS); Perfluoro-*n*-[1,2-^13^C]decanoic acid (MPFDA); perfluoro-n-[1,2,3,4,5,6-^13^C_6_]decanoic acid (M6PFDA); sodium perfluoro-1-[^13^C_8_]octanesulfonate (M8PFOS). Error bars represent standard deviation of three replicates.

**Figure S6**. Scoring of eye pigment (top) at hatch and body pigment (bottom) at hatch in fathead minnow fry exposed to “no tire” controls, 1-, 3- and 10-day filtered (F) and unfiltered (U) tire leachates for 6 days. Treatments are presented in replicates (N=3).

*** The scoring system was created by referring to a study for Yeongsan River

**Figure S7**. Average relative area (individual/cumulative area in sample) for (A) all peaks, (B) cyclic amines, (C) benzothiazoles identified in positive mode and (D) all peaks identified in negative mode. Peaks in (A) and (D) represent the average of all day 1-10 leachate samples.

**Table S1**. Retention time (RT), calibration curve range, regression coefficients (R^2^), and average response factors determined from internal standards triphenyl d_15_ phosphate (d_15_TPP) and tris(2-butoxyethyl)phosphate-d_27_ (_d27_TBEP) in positive mode. All compounds were detected are [M+H]^+^ with the exception of hexa(methoxymethly)melamine [M+Na]^+^.

| **Chemical** | **Exact Mass** | **Abbrv** | **RT** | **Cal. range** | **R2** | **Average response factors ± SD** | | **Log Kow** | **Solubility** |
| --- | --- | --- | --- | --- | --- | --- | --- | --- | --- |
|  |  |  | **(min)** | **(pg/µl)** |  | **d15TPP** | **d27TBEP** |  | **(mg/L)** |
| Benzotriazole | 119.04835 | BZTR | 4.42 | 10-1000 | 0.994 | 0.13± 0.02 | 0.20 ± 0.05 | 1.44 | 19800 |
| 5-Methyl-1H-Benzotriazole | 133.06400 | MBZTR | 5.44 | 10-500 | 0.990 | 0.64± 0.05 | 0.97 ± 0.18 | 1.71 | 3069 |
| Benzothiazole | 135.01427 | BT | 5.89 | 50-1000 | 1.000 | 0.13± 0.08 | 0.19 ± 0.11 | 2.01 | 4300 |
| 2-Mercaptobenzothiazole | 166.98634 | MBT | 6.01 | 50-500 | 0.968 | 0.0027±0.001 | 0.004±0.002 | 2.42 | 120 |
| N-phenyl-1,4-phenylenediamine | 184.10005 | PPDA | 6.10 | 10-1000 | 0.994 | 0.74 ± 0.21 | 1.08 ± 0.34 | 1.82 | 1450 |
| Hexa(methoxymethyl)melamine | 390.22268 | HMMM | 6.64 | 10-500 | 0.999 | 0.30± 0.05 | 0.46± 0.12 | 1.61 | 149.3 |
| Phenazine | 180.06875 | PHE | 6.99 | 10-500 | 0.981 | 1.44± 0.30 | 2.17 ± 0.54 | 2.84 | 16 |
| N,N'-Dicyclohexylurea | 224.18886 | DCU | 7.63 | 50-1000 | 0.959 | 0.0011 ± 0.0008 | 0.0016 ± 0.001 | 3.92 | 14.78 |
| 2-Methylthio-benzothiazole | 181.00199 | MeSBT | 7.72 | 10-1000 | 0.994 | 0.27 ± 0.04 | 0.40 ± 0.09 | 3.15 | 125 |
| Benzo[h]quinoline | 179.07350 | BhQ | 7.89 | 10-500 | 0.991 | 1.35 ± 0.11 | 2.04 ± 0.38 | 3.43 | 5.08 |
| 1,2-Dihydro-2,2,4-trimethylquinoline | 173.12045 | TMQ | 8.23 | 10-500 | 0.999 | 1.02 ± 0.30 | 1.53 ± 0.45 | 3.3 | 89.710 |
| N-cyclohexylbenzothiazole-2-sulfenamide | 264.07549 | CBS | 9.55 | 10-500 | 0.977 | 1.15 ± 0.24 | 1.74± 0.45 | 3.47 | 21.39 |
| Benzo[c]acridine | 229.08915 | BcAcr | 9.65 | 10-500 | 0.986 | 2.25± 0.39 | 3.37 ± 0.68 | 4.49 | 0.346 |

**Table S2.** Data for water quality measured during the embryo exposures of fathead minnow tire leachates and control waters. Table shows means and standard deviation (SD.). All tire leachate exposures were at 100 % concentration from 10 g tire particles per L. Data are means of 9 measurements for controls of lab water sourced from Lake Ontario water (particle filtered, carbon-filtered, and UV sterilized) and 3 measurements for leachate samples.

| **Treatment** | **Temperature (°C)** | **SD** | **Conductivity (µS/cm)** | **SD** | **pH** | **SD** | **Dissolved oxygen (mg/L)** | **SD** | **Free NH_3_ (mg/L)** | **SD** |
| --- | --- | --- | --- | --- | --- | --- | --- | --- | --- | --- |
| Controls | 23.9 | 0.1 | 368.0 | 7.8 | 8.12 | 0.02 | 8.30 | 0.04 | 0.011 | 0.015 |
| No Tire Filt | 24.1 | 0.05 | 355.8 | 5.0 | 7.99 | 0.02 | 7.95 | 0.10 | 0.000 | 0.000 |
| No Tire Unfilt | 24.0 | 0.05 | 346.8 | 1.4 | 7.99 | 0.01 | 8.08 | 0.06 | 0.003 | 0.005 |
| 1 Day Filt | 24.0 | 0.05 | 364.3 | 0.9 | 8.05 | 0.02 | 8.17 | 0.03 | 0.007 | 0.005 |
| 1 Day Unfilt | 24.1 | 0.08 | 362.9 | 1.3 | 8.08 | 0.01 | 8.14 | 0.01 | 0.010 | 0.000 |
| 3 Day Filt | 24.1 | 0.08 | 360.5 | 1.0 | 8.06 | 0.00 | 8.14 | 0.02 | 0.010 | 0.020 |
| 3 Day Unfilt | 24.1 | 0.05 | 361.4 | 1.0 | 8.04 | 0.01 | 8.01 | 0.02 | 0.023 | 0.020 |
| 10 Day Filt | 24.1 | 0.05 | 347.7 | 1.3 | 8.04 | 0.01 | 8.18 | 0.02 | 0.050 | 0.000 |
| 10 Day Unfilt | 24.1 | 0.1 | 345.3 | 0.1 | 8.09 | 0.03 | 8.25 | 0.04 | 0.083 | 0.024 |

**Table S3**. Estimated concentrations of organic chemicals tire particle (TP) and TP leachates in present study compared to previous studies (full names for abbreviated chemicals are given in Table S4).

|  |  |  |  |  |  |  |  |  |  |  |  |  |  |  |  |  |
| --- | --- | --- | --- | --- | --- | --- | --- | --- | --- | --- | --- | --- | --- | --- | --- | --- |
| **Abbrv.** | **Leachate concentration (ng/L)** | | | | |  |  | **Loading in TPs (µg/g TP)** | | | | |  |  | **Literature values** | |
|  | **Day 1** | **SD** | **Day 3** | **SD** | **Day 10** | **SD** |  | **Day 1** | **SD** | **Day 3** | **SD** | **Day 10** | **SD** |  | **Leachate (ng/L)** | **Tire or TRWPs (µg/g)** |
| BT | 143 | 31 | 22 | 59 | 263 | 117 |  | 0.014 | 0.003 | 0.025 | 0.006 | 0.026 | 0.012 |  | 2300000 (freshwater) and 1460000 (marine)^6^ | 20.6 - 175.2 (52.41)^1^; 37 - 105^3 ;^ 39-155 ^4^; 2.3-22^5;^ 29 (freshwater) and 18 (marine)^6^; 35±18^7^ |
| ABZ | 17 | 4.3 | 39 | 14.2 | 65 | 20.3 |  | 0.002 | 0.0004 | 0.004 | 0.001 | 0.007 | 0.002 |  |  |  |
| NHBT | 35 | 11 | 58 | 14 | 79 | 18 |  | 0.004 | 0.001 | 0.006 | 0.001 | 0.008 | 0.002 |  |  | 0.29 - 20.5 (12.6)^1^; 0.001 - 0.006^5^ |
| OHBT | 114 | 33 | 123 | 29 | 150 | 31 |  | 0.011 | 0.003 | 0.012 | 0.003 | 0.015 | 0.003 |  |  | 6.5 - 40.5 (19.1)^1^; 0.3 - 2.8^5^ |
| MBT | 249 | 160 | 298 | 101 | 465 | 88 |  | 0.025 | 0.016 | 0.030 | 0.010 | 0.047 | 0.009 |  |  | 3.8 - 35.9 (13.8)^1^; 53- 2170^4^; 0.1-34^5^; 7.5±3.5^7^ |
| DPA | 15 | 8.2 | 8 | 1.8 | 10 | 2.4 |  | 0.002 | 0.001 | 0.001 | 0.0003 | 0.001 | 0.0002 |  |  |  |
| TMQ | 26 | 17 | 28 | 15 | 36 | 15 |  | 0.003 | 0.002 | 0.003 | 0.002 | 0.004 | 0.002 |  |  |  |
| ACT | 13 | 4.2 | 10 | 2.8 | 16 | 5.2 |  | 0.001 | 0.0004 | 0.001 | 0.000 | 0.002 | 0.001 |  |  |  |
| ACR | 0.95 | 0.24 | 0.96 | 0.25 | 1.2 | 0.26 |  | 0.00009 | 0.00002 | 0.0001 | 0.00002 | 0.0001 | 0.00003 |  |  |  |
| MeSBT | 12 | 4.5 | 19 | 6.4 | 34 | 12 |  | 0.001 | 0.000 | 0.002 | 0.001 | 0.003 | 0.001 |  |  | 0.16 - 1.9 (0.75)^1^; 0.80 - 2.70^4^; 0.11 -0.26^5^; <4^7^ |
| DHA | 23298 | 9861.0 | 39708 | 13401.9 | 31786 | 10294.4 |  | 2.330 | 0.986 | 3.971 | 1.340 | 3.179 | 1.029 |  |  |  |
| PPDA | 1.8 | 0.74 | 2.0 | 0.43 | 4.8 | 2.2 |  | 0.0002 | 0.00007 | 0.0002 | 0.00004 | 0.0005 | 0.0002 |  |  |  |
| NDPA | 1.8 | 0.6 | 2.2 | 0.5 | 1.6 | 0.5 |  | 0.0002 | 0.00006 | 0.0002 | 0.00002 | 0.0002 | 0.00005 |  |  |  |
| DPG | 7900 | 2300 | 12000 | 2700 | 13000 | 2700 |  | 0.79 | 0.23 | 1.21 | 0.27 | 1.30 | 0.27 |  | 99 - 28400^2^ | 100±16 ^2;^ 310±21^7^ |
| DPU | 2400 | 510 | 3400 | 832 | 3796 | 806 |  | 0.24 | 0.05 | 0.34 | 0.08 | 0.38 | 0.08 |  |  |  |
| CPU | 5068 | 1180 | 5690 | 1443 | 6059 | 1382 |  | 0.50 | 0.12 | 0.57 | 0.14 | 0.60 | 0.14 |  |  |  |
| DHU | 397000 | 10000 | 440000 | 10300 | 450000 | 96000 |  | 40 | 10 | 44 | 10 | 45 | 9.6 |  | 20-5130^2^ | 18±2^2^ |
| PBI | 32 | 8.8 | 28 | 6.5 | 36 | 7.9 |  | 0.003 | 0.001 | 0.003 | 0.001 | 0.004 | 0.001 |  |  |  |
| NCBA | 6.6 | 1.8 | 7 | 1.5 | 7 | 1.7 |  | 0.001 | 0.0002 | 0.001 | 0.000 | 0.001 | 0.000 |  |  | 2^7^ |
| DHPA | 0.14 | 0.04 | 0.47 | 0.1 | 1.1 | 0.3 |  | 0.00001 | 0.000 | 0.00005 | 0.00001 | 0.0001 | 0.00003 |  |  |  |
| HMMM | 0.3 | 0.1 | 1.1 | 0.3 | 0.4 | 0.1 |  | 0.00003 | 0.00001 | 0.0001 | 0.00003 | 0.00004 | 0.00001 |  | 24-4750^2^ | 23±2^2^ |

[1] Concentrations in 17 tires, range (average) (Zhang et al. 2018); [2] Leachate conc range (1-250 mg/L TWP concentration); TWP (µg/g) is the loading is average of leachate concentration across loadings (Peter et al. 2020); [3] Crumb tire rubber (Halsband et al. 2020); [4] Mean and (SD) in different makes of tires, (n=3 for each), only means reported in table (Avagyan et al. 2014); [5] Assessed 7 tires for benzothiazoles and benzotriazoles (Asheim et al. 2019); [6] Car tire rubber derived freshwater and marine leachates, prepared from 80 g/L (25 C, 14 d) (Capolupo et al. 2020); [7] Fresh Tire wear and road wear generated particles (Unice et al. 2015).

**Table S4.** Summary of two-way ANOVA results using factors days of leaching and filter for the toxicity endpoints studied.

| **Endpoint** | **Factor** | **Sum Sq** | **Df** | **F-value** | **Pr(>F)** |
| --- | --- | --- | --- | --- | --- |
| Time to hatch | Days | 0.098 | 3 | 0.4778 | 0.70 |
|  | Filter | 0.102 | 1 | 1.4871 | 0.24 |
|  | Days : Filter | 0.08 | 3 | 0.3895 | 0.76 |
|  | Residuals | 1.094 | 16 |  |  |
|  |  |  |  |  |  |
| Heart rate | Days | 475 | 3 | 5.145 | 0.01 |
|  | Filter | 26 | 1 | 0.8526 | 0.37 |
|  | Days : Filter | 571 | 3 | 6.182 | 0.01 |
|  | Residuals | 431 | 14 |  |  |
|  |  |  |  |  |  |
| Hatchability | Days | 0.0165 | 3 | 1.7156 | 0.20 |
|  | Filter | 0.0001 | 1 | 0.0258 | 0.87 |
|  | Days : Filter | 0.0017 | 3 | 0.1784 | 0.91 |
|  | Residuals | 0.0513 | 16 |  |  |
|  |  |  |  |  |  |
| Hatch success | Days | 9206.3 | 3 | 15.4496 | 0.00 |
|  | Filter | 1623.1 | 1 | 8.1715 | 0.01 |
|  | Days : Filter | 3135.2 | 3 | 5.2615 | 0.01 |
|  | Residuals | 3178.1 | 16 |  |  |
|  |  |  |  |  |  |
| Length at hatch | Days | 0.49 | 3 | 20.2139 | 0.00 |
|  | Filtration | 0.004 | 1 | 0.4894 | 0.49 |
|  | Days:Filtration | 0.146 | 3 | 6.0285 | 0.01 |
|  | Residuals | 0.129 | 16 |  |  |
|  |  |  |  |  |  |
| Hatch severity | Days | 7536 | 3 | 12.2067 | 0.00 |
|  | Filter | 1755.5 | 1 | 8.5307 | 0.01 |
|  | Days : Filter | 3344.5 | 3 | 5.4173 | 0.01 |
|  | Residuals | 3292.6 | 16 |  |  |

**Table S5.** Chemical formula, exact mass of precursor ions and 100 most intense product ions observed of studied compounds.

| **Compound** | **Chemical Formula** | **Structure** | **Precursor ion and accurate mass (m/z)** | **Product ions** | **Error (ppm)** | **Basis for identification (Level of identifications)** |
| --- | --- | --- | --- | --- | --- | --- |
| Benzothiazole  (BT) | C_7_H_5_NS | 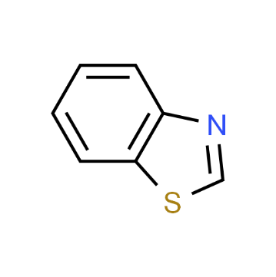 | 136.02168  [M+H]  135.01448 | 109.01066  105.04475  95.04913  77.03858  68.97945  65.03874 | 1.10 (5.5) | Reference standard confirmation  (Level 1) |
| 2-Mercaptobenzothiazole  (MBT) | C_7_H_5_NS_2_ | 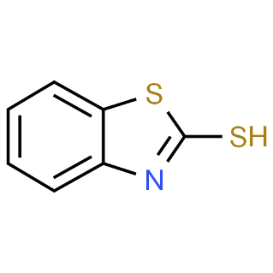 | 167.99388  [M+H]  166.98655 | 150.12865  134.77278  86.06001  83.08553  58.06541  55.05466 | 0.70 (5.5) | Reference standard confirmation, mzCloud match 92%.  (Level 1) |
| 1,2-Dihydro-2,2,4-trimethylquinoline  (TMQ) | C_12_H_15_N | 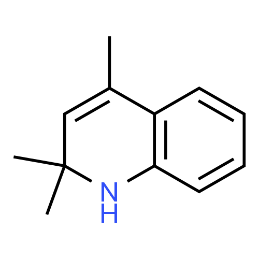 | 174.12790  [M+H]  173.12060 | 158.09639  144.08075  130.06512  117.05727  106.06507  91.05425 | 2.14 (5.5) | Reference standard confirmation  (Level 1) |
| 2-(Methylthio)benzothiazole  (MeSBT) | C_8_H_7_NS_2_ | 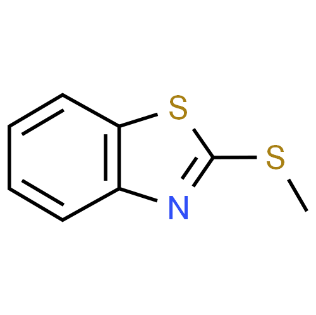 | 182.00945  [M+H]  181.00215 | 166.98578  135.01373  123.01367  109.01068  91.04173 | 1.72 (5.5) | Reference standard confirmation  (Level 1) |
| N-Phenyl-1,4 benzenediamine  (PPDA) | C_12_H_12_N_2_ | 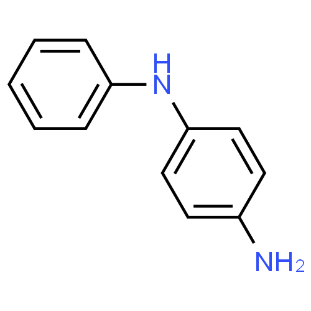 | 185.10765  [M+H]  184.10004 | 167.07301  108.06820  93.05737  81.05736 | -1.43 (7.5) | Reference standard confirmation  (Level 1) |
| N,N'-Dicyclohexylurea  (DHU) | C_13_H_24_N_2_O | 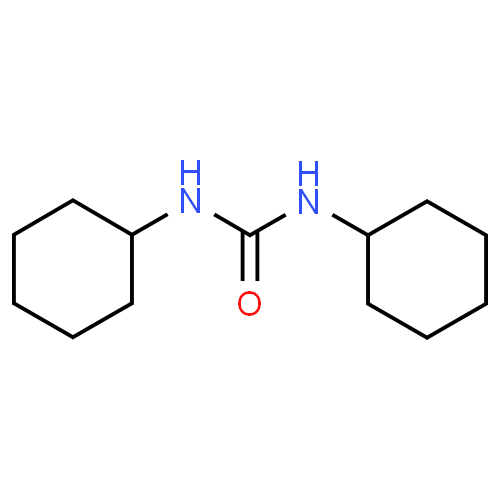 | 225.19646  [M+H]  224.18903 | 191.33096  143.11787  100.11205  83.08552  61.03989  55.05458 | 1.42 (2.5) | Reference standard confirmation, mzCloud match 98%.  (Level 1) |
| Hexa(methoxymethyl) melamine  (HMMM) | C_15_H_30_N_6_O_6_ | 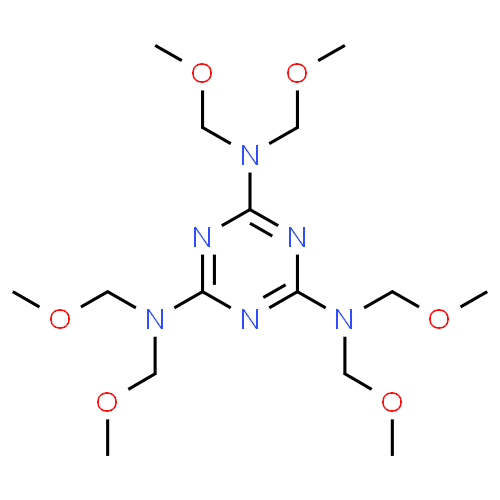 | 413.21194  [M+Na]  390.22660 |  | 0.40 (3.5) | RT and accurate mass reference standard confirmation  (Level 1) |
| **Level 2- 4 Compounds** | | | | | | |
| 3-Aminobenzamide  (ABZ) | C_7_H_8_N_2_O | 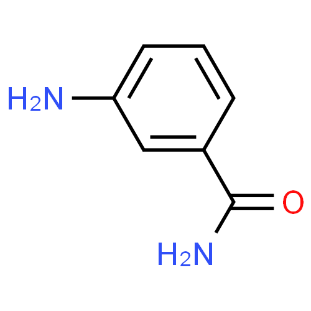 | 137.07108  [M+H]  136.06379 | 105.04472  95.04911  94.06512  93.05730  77.03855 | 0.90 (4.5) | mzCloud match, 90%.  (Level 2) |
| 2-Aminobenzothiazole  (NHBT) | C_7_H_6_N_2_S | 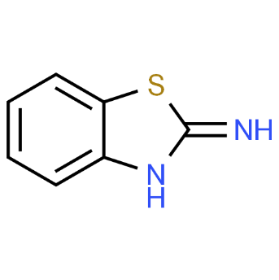 | 151.03281  [M+H]  150.02555 | 124.02158  122.00583  109.01067  96.00282  92.04946  80.04947  65.03875 | 2.8 (5.5) | mzCloud match, 83%.  (Level 2) |
| 2-Hydroxybenzothiazole  (OHBT) | C_7_H_5_NOS | 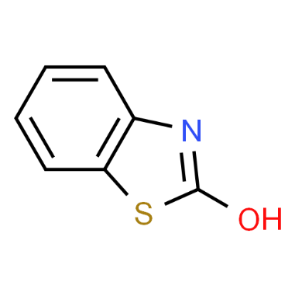 | 152.01682  [M+H]  151.00950 | 124.02153  119.03653  109.01064  97.01067  92.04945  91.04162  90.03384  80.04945  65.03873 | 2.6 (5.5) | mzCloud match, 84%.  (Level 2) |
| P1 | C_11_H_11_N |  | 158.09650  [M+H]  157.08947 | 143.07294  115.05422  91.05425 | 0.47 (6.5) | MS/MS but no match.  (Level 3)  All metfrag matches have an aromatic ring |
| Diphenylamine  (DPA) | C_12_H_11_N | 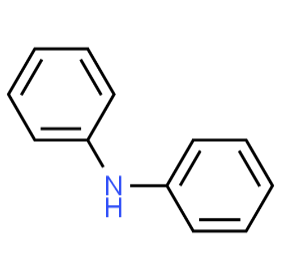 | 170.09665  [M+H]  169.08944 | 165.84460  152.06210  93.05738  92.04942  65.03873  56.96503 | 1.31 (7.5) | mzCloud match, 91%.  (Level 2) |
| Acetoacetanilide  (ACT) | C_10_H_11_NO_2_ |  | 178.086618 [M+H]  177.07932 | 136.07574  120.04435  105.04476  94.06516 | 2.05 (5.5) | mzCloud match, 75%.  (Level 2) |
| Acridine  (ACR) | C_13_H_9_N | 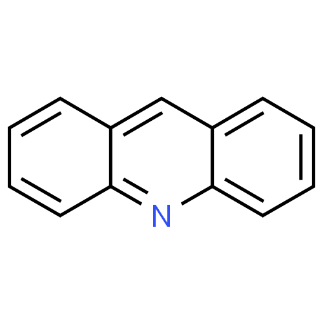 | 180.08099 [M+H]  179.07371 | 152.06027  179.07303  77.03860 | 1.19 (9.5) | mzCloud match, 80%.  Similar fragmentation to authentic standard benzo[h]quinolone but RTs do not match.  (Level 2) |
| Dicyclohexylamine  (DHA) | C_12_H_23_N |  | 182.19072  [M+H]  181.18349 | 100.11200  83.08552  55.05457 | 2.20 (1.5) | MS/MS but no match.  Similar fragments to N,N’-dicycohexylurea. Also reported as Dicyclohexylamine by Seiwart et al. 2018.  (Level 2) |
| P2 | C_12_H_9_NO |  | 184.07571  [M+H]  183.06870 | 183.06808  155.06035  154.06508  129.06984  128.06204  117.05733  105.04476  95.04915  77.03859 | 0.11 (8.5) | MS/MS but no match.  (Level 4) |
| P3 | C_12_H_11_NO |  | 186.09151  [M+H]  185.08413 | 167.07826  141.06978  109.05225  93.05736  92.04948  81.03358  80.04949  65.03878 | 0.91 (7.5) | MS/MS but no match.  (Level 4) |
| P4 | C_11_H_13_NO_2_ |  | 192.10194  [M+H]  191.09470 | 174..09918  150.09134  149.08354  134.06001  108.08077 | 0.70 (5.5) | MS/MS but no match.  (Level 4) |
| P5 | C_13_H_10_N_2_ |  | 195.09203  [M+H]  194.08445 | 92.04948  77.03875  65.03876 | -1.82 (9.5) | mzCloud match.  2-Phenylbenzimidazole (83%)  (Level 2) |
| 4-Nitrosodiphenylamine  (NDPA) | C_12_H_10_ N_2_O |  | 199.08693  [M+H]  198.0796 | 181.07600  169.08836  143.11787  130.06529  83.08556  61.03990 | 1.71 (8.5) | mzCloud match, 78%.  4-Nitrosodiphenylamine-Metfrag-KEGG  (Level 2) |
| N,N'-Diphenylguanidine  (DPG) | C_13_H_13_N_3_ | 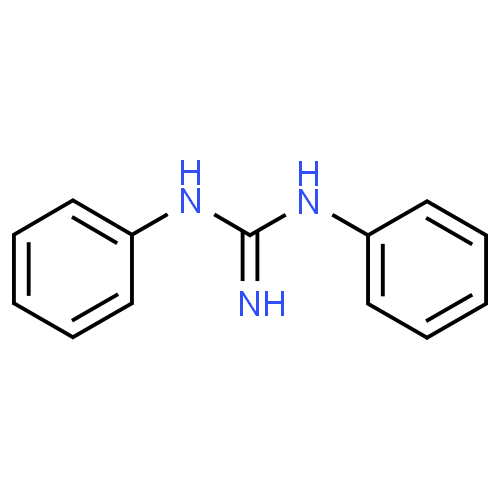 | 212.11818  [M+H]  211.11086 | 195.09174  119.06042  94.06516  77.03858 | -0.21 (8.5) | mzCloud spectral match 95%, and (Peters et al., 2018) Literature match.  (Level 2) |
| N,N'-Diphenylurea  (DPU) | C_13_H_12_N_2_O | 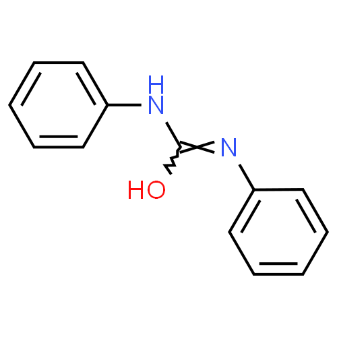 | 213.10254 [M+H]  212.09513 | 120.04443  105.04478  94.06518  92.04950  77.03860 | 1.41 (8.5) | mzCloud spectral match 90%  (Level 2) |
| 1-Cyclohexyl-3-phenylurea  (CPU) | C_13_H_18_N_2_O | 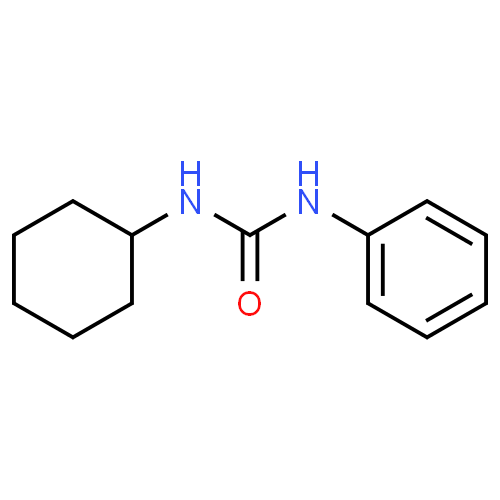 | 219.14941  [M+H]  218.14204 | 137.07095  120.04433  94.06512  83.08550  77.03858  55.05455 | 1 (5.5) | Literature match (Peters et al., 2018)  (Level 2) |
| 3-Phenyl-1,3-benzothiazol-2-imine  (PBI) | C_13_H_10_N_2_S | 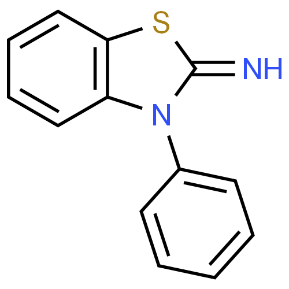 | 227.06403  [M+H]  226.05694 | 202.80905  194.08368  149.01657  124.02161  118.05238  109.01062  93.0573  92.04945  80.04948  65.03867 | 1.25 (9.5) | mzCloud spectral match 90%.  (Level 2) |
| P6 | C_14_H_14_N_2_O |  | 227.11801  [M+H]  226.11074 |  | 0.40 (8.5) | No MS/MS.  (Level 4) |
| P7 | C_18_H_25_NO |  | 227.21000  [M+H]  226.20477 |  | 1.02 (6.5) | No MS/MS.  (Level 4) |
| [P](https://www.ncbi.nlm.nih.gov/pcsubstance/?term=%224-Methyl-a-pyrrolidinobutiophenone%22%5bCompleteSynonym%5d%20AND%2057486975%5bStandardizedCID%5d)8 | C_15_H_21_N O |  | 232.16989  [M+H]  231.16255 | 174.16727  159.10429  158.09644  132.08072  118.06506 | 1.30 (5.5) | MS/MS but no match.  (Level 4) |
| N-Cyclohexyl-1,3-benzothiazol-2-amine (NCBA) | [C_13_H_16_N_2_S](https://pubchem.ncbi.nlm.nih.gov/#query=C13H16N2S) |  | 233.11052  [M+H]  232.10363 | 151.0324  124.02155  109.01072  92.04952  83.08552  55.05458 | -0.75 (6.5) | On GAD list |
| P9 | C_12_H_12_O_4_N  C_13_H_8_N_5_ |  | 235.0843 [M+H]  234.077001 |  | 2.1 (7.5)  -3.6 (12) | No MS/MS.  (Level 4) |
| 4,5-diphenyl-1H-pyrazol-3-amine  (DHPA) | C_15_H_13_ N_3_ | 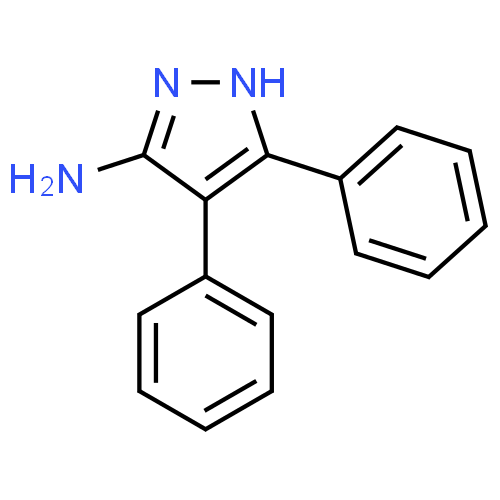 | 236.11860  [M+H]  235.11130 | 158.07170  133.07597  118.06512  106.06511  91.05427 | 1.1 (10.5) | mzCloud spectral match 82%.  (Level 2) |
| P10 |  |  | 241.1311 [M+H]  240.12380 |  |  | No MS/MS.  (Level 4) |
| P11 | C_15_H_15_NO_2_ |  | 242.11787  [M+H]  241.10994 | 224.10683  209.08353  185.08353  180.08072  156.08076  92.04948 | 1.3 (8.5) | MS/MS but no match.  (Level 4) |
| P12 |  |  | 247.1782 [M+H]  246.170901 |  |  | No MS/MS.  (Level 4) |
| P13 |  |  | 249.10007  [M+H]  248.09627 |  |  | No MS/MS.  (Level 4) |
| P14 | C_18_H_14_N_2_ |  | 259.12334  [M+H]  258.11619 | 182.08401  181.07593  167.07289  156.08083  154.06505  93.05726  92.04945 | 1.56 (12.5) | MS/MS but no match.  (Level 4) |
| P15 | C_16_H_13_N_3_O |  | 264.11316  [M+H]  263.10609 | 236.11807  219.09171  207.09181  195.09169  158.07124  143.06041  133.07607  118.06509  106.06512  104.04948  91.05422 | 0.42 (11.5) | MS/MS but no match.  (Level 4) |
| P16 | C_15_H_15_N_3_O |  | 266.12869  [M+H]  265.12160 | 195.09169  145.07603  120.08078  92.04948 | 0.34 (10.5) | MS/MS but no match.  Unidentified peak in Seiwart et al (2020) associated with DPA.  (Level 3) |
| P17 | C_18_H_22_N_2_ |  | 267.1859 [M+H]  266.17860 |  | 2.3 (8.5) |  |
| P18 | C_18_H_25_NO |  | 272.20065  [M+H]  271.19361 | 242.15410  215.16684  214.15891  199.13553  174.12776  158.09641  132.08078 | -0.88 (6.5) | MS/MS but no match.  (Level 4) |
| P19 | C_16_H_15_N_3_O_2_ |  | 282.12387  [M+H]  281.11679 | 264.11295  212.11818  195.09174  161.07092  136.07571  119.06041  94.06516  92.04950  60.4465 | 0.6 (10.5) | MS/MS but no match.  (Level 4) |
| P20 | C_19_H_17_N_3_ | 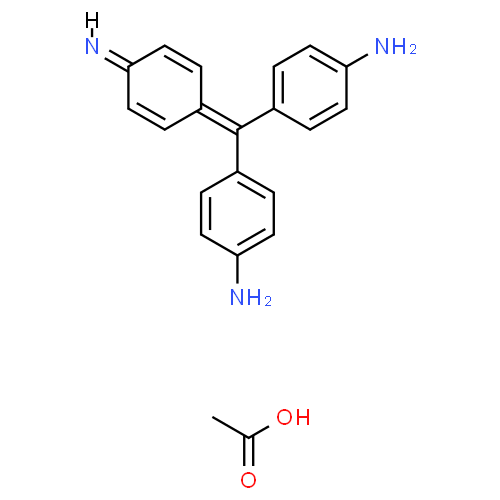 | 288.14975  [M+H]  287.14235 | 230.15376  195.09174  168.08098  117.04463  94.06515  92.04949 | 0.37 (12.5) | mzCloud spectral match HAS 3 HITS: Highest for  Basic Red (77%) |
| P21 |  |  | 294.16061  [M+H]  293.15308 | 212.11807  195.09161  119.06036  119.06036  94.06513  72.08080 |  | MS/MS but no match.  (Level 4) |
| P22 |  |  | 295.18091  [M+H]  294.17363 | 239.11775  174.12767  159.10413  144.08067  132.08075  118.06509  106.06506 |  | MS/MS but no match.  (Level 4) |
| P23 |  |  | 306.16058  [M+H]  305.15297 | 278.16589  195.09172  84.08082 |  | MS/MS but no match.  (Level 4) |
| P24 |  |  | 311.16754  [M+H]  310.16022 |  |  | No MS/MS.  (Level 3) |
| P25 |  |  | 317.18619  [M+H]  316.17885 | 259.14395  244.12062  243.11253  186.07893  91.75128 |  | MS/MS but no match.  (Level 4) |
| P26 |  |  | 324.17099  [M+H]  323.16352 | 306.16003  264.14914  212.11815  195.09164  148.07562  119.06038  94.06518 |  | MS/MS but no match.  (Level 4) |
| P27 |  |  | 330.24301  [M+H]  329.23574 | 272.20087  215.16675  214.15894  199.13457  174.12769  123.08033  94.06517 |  | MS/MS but no match.  (Level 4) |
| P28 |  |  | 331.217028 [M+H]  330.20973 | 316.19321  301.16992  174.12766  171.10413  158.09644 | 0.77 (11.5) | mzCloud spectral match HAS 3 HITS: Highest for:  Leucomalachite green (80%)  (Level 3) |
| P29 |  |  | 437.29120  [M+H]  436.28370 | 219.14925  137.07108  94.06521 |  | MS/MS but no match.  (Level 4) |
| **ESI NEGATIVE** | | | | | | |
| N1 |  |  | 148.05113  [M-H]  149.05898 |  |  | No MS/MS  Level 4 |
| N2 |  |  | 185.15320  [M-H]  186.16105 |  |  | No MS/MS  Level 4 |
| N3 |  |  | 193.08884  [M-H]  194.09668 | 79.95561 |  | No MS/MS  Level 4 |
| N4 |  |  | 213.96252  [M-H]  214.97037 |  |  | No MS/MS  Level 4 |
| N5 |  |  | 214.04951  [M-H]  215.05736 |  |  | No MS/MS  Level 4 |
| N6 |  |  | 214.08580  [M-H]  215.05736 |  |  | No MS/MS  Level 4 |
| N7 |  |  | 216.06506  [M-H]  215.05736 |  |  | No MS/MS  Level 4 |
| N8 |  |  | 217.09764  [M-H]  218.10475 | 192.97442  120.96021  111.00690  99.00700  92.04877 |  | No MS/MS Library Match  Level 4 |
| N9 |  |  | 218.16632  [M-H]  219.17378 |  |  | No MS/MS  Level 4 |
| N10 |  |  | 220.14551  [M-H]  221.15335 |  |  | No MS/MS  Level 4 |
| N11 |  |  | 221.08052  [M-H]  222.08837 |  |  | No MS/MS  Level 4 |
| N12 |  |  | 225.19617  [M-H]  226.20355 |  |  | No MS/MS  Level 4 |
| N13 |  |  | 227.08116  [M-H]  228.08901 |  |  | No MS/MS  Level 4 |
| N14 |  |  | 228.06528  [M-H]  229.07312 |  |  | No MS/MS  Level 4 |
| N15 |  |  | 233.12831  [M-H]  234.13616 | 202.10991  174.11501  108.04375 |  | No MS/MS Library Match  Level 4 |
| N16 |  |  | 235.16911  [M-H]  221.15335 |  |  | No MS/MS  Level 4 |
| N17 |  |  | 239.12531  [M-H]  240.13315 |  |  | No MS/MS  Level 4 |
| N18 |  |  | 244.09671  [M-H]  245.10456 |  |  | No MS/MS  Level 4 |
| N19 |  |  | 245.93484  [M-H]  246.94269 | 165.97752  134.00523  180.04112 |  | No MS/MS Library Match  Level 4 |
| N20 |  |  | 247.06326  [M-H]  248.07111 | 101.02278  92.04887  73.02792 |  | No MS/MS Library Match  Level 4 |
| N21 |  |  | 250.13139  [M-H]  251.13924 |  |  | No MS/MS  Level 4 |
| N22 |  |  | 251.16492  [M-H]  252.17276 | 92.04878  71.01211 |  | No MS/MS Library Match  Level 4 |
| N23 |  |  | 253.11020  [M-H]  254.11805 | 227.82921  103.00185  85.02766  71.01210  59.01216 |  | No MS/MS Library Match  Level 4 |
| N24 |  |  | 256.07196  [M-H]  257.11537 |  |  | No MS/MS  Level 4 |
| N25 |  |  | 256.17847  [M-H]  257.18631 |  |  | No MS/MS  Level 4 |
| N26 |  |  | 257.09240  [M-H]  258.10025 | 92.04878  75.00700  105.01767 |  | No MS/MS Library Match  Level 4 |
| N27 |  |  | 264.07703  [M-H]  265.08487 |  |  | No MS/MS  Level 4 |
| N28 |  |  | 265.15488  [M-H]  266.16227 |  |  | No MS/MS  Level 4 |
| N29 |  |  | 269.18643  [M-H]  270.19428 |  |  | No MS/MS  Level 4 |
| N30 |  |  | 286.13394  [M-H]  287.14130 |  |  | No MS/MS  Level 4 |
| N31 |  |  | 295.05710  [M-H]  296.06495 |  |  | No MS/MS  Level 4 |
| N32 |  |  | 299.20081  [M-H]  300.20792 |  |  | No MS/MS  Level 4 |
| N33 |  |  | 305.11743  [M-H]  306.12487 |  |  | No MS/MS  Level 4 |
| N34 |  |  | 309.15164  [M-H]  310.15949 |  |  | No MS/MS  Level 4 |
| N35 |  |  | 320.13910  [M-H]  321.14658 |  |  | No MS/MS  Level 4 |
| N36 |  |  | 325.14645  [M-H]  326.15430 |  |  | No MS/MS  Level 4 |
| N37 |  |  | 347.14218  [M-H]  348.15003 | 262.04092  209.10666  79.95559 |  | No MS/MS Library Match  Level 4 |
| N38 |  |  | 349.04623  [M-H]  350.05408 |  |  | No MS/MS  Level 4 |
| N39 |  |  | 358.15469  [M-H]  359.16254 |  |  | No MS/MS  Level 4 |
| N40 |  |  | 367.18967  [M-H]  368.19751 | 177.09055  268.11087 |  | No MS/MS Library Match  Level 4 |
| N41 |  |  | 384.18039  [M-H]  385.18824 | 194.08073  177.09062  92.04879 |  | No MS/MS Library Match  Level 4 |
| N42 |  |  | 395.18637  [M-H]  396.19422 | 302.17259  249.08975  201.10175  171.05484  117  04408  92.04875 |  | No MS/MS Library Match  Level 4 |
| N43 |  |  | 429.00336  [M-H]  430.01120 |  |  | No MS/MS  Level 4 |
| N44 |  |  | 529.46130  [M-H]  530.46915 |  |  | No MS/MS  Level 4 |

**Table S6**. Principal Component Analysis loadings of all peak features in leachate samples.

|  | PC1 | PC2 | PC3 | PC4 | PC5 |  |  | PC1 | PC2 | PC3 | PC4 | PC5 |
| --- | --- | --- | --- | --- | --- | --- | --- | --- | --- | --- | --- | --- |
| Eigenvalue | 43.8 | 24.8 | 15.5 | 7.1 | 3.7 |  |  |  |  |  |  |  |
| Cumulative | 43.8 | 68.6 | 84.2 | 91.3 | 95 |  |  |  |  |  |  |  |
| **Positive Mode** | | | | | |  | **Negative mode** | | | | | |
| BT | -0.43 | -0.28 | 0.26 | 0.40 | 0.68 |  | N1 | 0.02 | 0.27 | -0.86 | 0.30 | 0.13 |
| ABZ | -0.82 | -0.24 | 0.38 | 0.19 | 0.21 |  | N2 | -0.89 | -0.32 | 0.17 | 0.18 | -0.20 |
| NHBT | -0.83 | -0.17 | 0.42 | 0.14 | -0.11 |  | N3 | 0.82 | 0.07 | 0.14 | 0.49 | -0.21 |
| OHBT | -0.34 | 0.68 | 0.56 | 0.27 | -0.19 |  | N4 | 0.18 | 0.02 | 0.64 | -0.17 | 0.62 |
| P1 | -0.22 | -0.18 | -0.35 | 0.89 | 0.02 |  | N5 | 0.84 | -0.35 | 0.30 | 0.18 | -0.10 |
| MBT | -0.82 | -0.07 | 0.55 | -0.04 | 0.04 |  | N6 | 0.97 | -0.06 | 0.20 | -0.01 | 0.02 |
| DPA | 0.38 | 0.70 | -0.09 | 0.57 | -0.10 |  | N7 | 0.97 | -0.06 | 0.20 | 0.01 | 0.02 |
| TMQ | 0.03 | 0.33 | -0.61 | 0.23 | 0.64 |  | N8 | 0.88 | 0.44 | -0.10 | -0.08 | 0.12 |
| ACT | -0.26 | 0.87 | -0.01 | 0.10 | 0.20 |  | N9 | 0.84 | -0.32 | -0.11 | 0.37 | -0.12 |
| ACR | -0.22 | 0.78 | 0.56 | 0.01 | 0.05 |  | N10 | 0.83 | -0.40 | 0.00 | 0.27 | -0.16 |
| MeSBT | -0.79 | -0.18 | -0.13 | 0.51 | 0.23 |  | N11 | -0.89 | -0.40 | 0.16 | 0.10 | -0.05 |
| DHA | 0.05 | -0.52 | -0.12 | 0.71 | 0.35 |  | N12 | 0.77 | -0.41 | 0.28 | 0.33 | -0.08 |
| P2 | 0.60 | 0.59 | 0.44 | 0.22 | -0.12 |  | N13 | 0.12 | -0.71 | 0.67 | -0.03 | 0.09 |
| PPDA | -0.68 | 0.21 | 0.03 | 0.51 | 0.35 |  | N14 | 0.96 | -0.05 | 0.23 | -0.02 | 0.04 |
| P3 | 0.40 | 0.82 | 0.20 | 0.35 | -0.03 |  | N15 | 0.78 | -0.13 | 0.32 | -0.38 | 0.07 |
| P4 | 0.65 | 0.74 | 0.08 | 0.08 | -0.05 |  | N16 | 0.97 | -0.11 | 0.02 | 0.15 | -0.03 |
| P5 | -0.49 | 0.26 | 0.29 | 0.43 | 0.54 |  | N17 | 0.16 | -0.75 | 0.53 | -0.16 | -0.02 |
| NDPA | 0.74 | 0.03 | 0.44 | 0.44 | 0.26 |  | N18 | 0.97 | -0.07 | 0.20 | -0.02 | 0.08 |
| DPG | -0.62 | -0.26 | 0.61 | 0.35 | -0.21 |  | N19 | -0.21 | -0.75 | 0.53 | 0.12 | -0.07 |
| DPU | -0.56 | 0.01 | 0.71 | -0.36 | 0.11 |  | N20 | 0.95 | -0.03 | 0.28 | -0.02 | 0.09 |
| CPU | -0.02 | 0.58 | 0.78 | 0.00 | 0.19 |  | N21 | 0.97 | -0.05 | 0.23 | -0.03 | 0.01 |
| DHU | 0.16 | 0.66 | 0.72 | 0.08 | -0.04 |  | N22 | 0.89 | 0.31 | 0.00 | -0.33 | 0.03 |
| PBI | -0.04 | 0.96 | 0.19 | 0.06 | -0.12 |  | N23 | 0.86 | 0.02 | 0.44 | -0.20 | 0.09 |
| P6 | -0.29 | -0.85 | 0.41 | 0.10 | -0.13 |  | N24 | 0.71 | -0.62 | -0.05 | 0.24 | -0.06 |
| P7 | -0.67 | 0.11 | 0.71 | -0.04 | 0.08 |  | N25 | 0.96 | -0.06 | 0.17 | -0.07 | -0.06 |
| P8 | 0.12 | 0.65 | 0.50 | 0.55 | -0.10 |  | N26 | 0.96 | -0.05 | 0.23 | -0.04 | 0.06 |
| NCBA | 0.22 | 0.81 | 0.43 | 0.20 | 0.16 |  | N27 | 0.90 | 0.04 | -0.35 | -0.17 | -0.06 |
| P9 | 0.35 | 0.86 | 0.37 | -0.09 | 0.00 |  | N28 | 0.52 | -0.65 | 0.52 | -0.06 | 0.06 |
| DHPA | -0.91 | -0.34 | 0.20 | 0.02 | 0.02 |  | N29 | 0.92 | -0.02 | 0.32 | -0.08 | 0.09 |
| P10 | 0.40 | 0.72 | 0.45 | 0.12 | 0.25 |  | N30 | 0.85 | -0.16 | -0.17 | 0.37 | 0.07 |
| P11 | 0.30 | -0.10 | -0.54 | 0.63 | -0.43 |  | N31 | 0.81 | -0.50 | 0.29 | 0.00 | 0.00 |
| P12 | 0.35 | 0.77 | 0.50 | -0.09 | 0.06 |  | N32 | 0.98 | -0.15 | -0.06 | 0.01 | -0.03 |
| P13 | -0.34 | -0.84 | 0.40 | 0.05 | -0.11 |  | N33 | -0.45 | 0.41 | 0.43 | 0.47 | -0.37 |
| P14 | 0.28 | 0.86 | 0.38 | 0.05 | -0.02 |  | N34 | 0.65 | -0.26 | 0.37 | -0.28 | 0.26 |
| P15 | -0.12 | 0.88 | -0.06 | 0.09 | -0.10 |  | N35 | -0.89 | 0.30 | 0.24 | 0.13 | -0.16 |
| P16 | 0.97 | 0.20 | -0.04 | 0.02 | 0.05 |  | N36 | 0.31 | -0.81 | 0.49 | 0.07 | -0.05 |
| P17 | 0.85 | 0.44 | -0.08 | -0.10 | 0.06 |  | N37 | -0.56 | 0.69 | 0.06 | -0.25 | 0.10 |
| P18 | -0.15 | -0.48 | 0.68 | -0.03 | 0.22 |  | N38 | -0.65 | -0.64 | 0.32 | 0.09 | 0.02 |
| P19 | 0.86 | 0.25 | 0.06 | 0.42 | 0.00 |  | N39 | 0.17 | -0.82 | 0.51 | -0.08 | -0.04 |
| P20 | 0.77 | -0.39 | 0.47 | -0.03 | -0.06 |  | N40 | 0.48 | 0.62 | 0.16 | 0.26 | -0.29 |
| P21 | 0.62 | -0.55 | 0.54 | 0.00 | -0.06 |  | N41 | -0.93 | 0.23 | 0.06 | 0.21 | -0.13 |
| P22 | -0.57 | 0.10 | 0.76 | 0.09 | -0.21 |  | N42 | 0.88 | -0.14 | -0.17 | 0.08 | -0.13 |
| P23 | -0.14 | 0.57 | 0.71 | 0.04 | -0.33 |  | N43 | 0.94 | -0.08 | 0.13 | 0.23 | 0.14 |
| P24 | -0.50 | 0.84 | -0.07 | -0.14 | 0.09 |  | N44 | -0.72 | -0.61 | 0.26 | 0.14 | -0.05 |
| P25 | 0.90 | 0.07 | -0.22 | 0.16 | -0.22 |  | N45 | -0.91 | -0.10 | -0.10 | 0.02 | -0.12 |
| P26 | -0.50 | -0.16 | 0.39 | 0.21 | -0.03 |  |  |  |  |  |  |  |
| P27 | 0.09 | 0.83 | 0.24 | -0.45 | -0.08 |  |  |  |  |  |  |  |
| P28 | -0.44 | 0.47 | 0.72 | 0.15 | -0.17 |  |  |  |  |  |  |  |
| HM | 0.43 | -0.79 | 0.42 | 0.08 | -0.08 |  |  |  |  |  |  |  |
| P29 | -0.50 | 0.48 | 0.47 | -0.45 | -0.20 |  |  |  |  |  |  |  |

**Table S7.** Eigenvalues and percentage of variance explained by the redundancy analysis (RDA).

| **RDA** | **Axis 1** | **Axis 2** | **Axis 3** | **Axis 4** | **Axis 5** | **Axis 6** | **Axis 7** |
| --- | --- | --- | --- | --- | --- | --- | --- |
| Eigenvalues | 7.67 | 5.32 | 3.24 | 1.83 | 0.43 | 0.33 | 0.15 |
| Proportion Explained | 0.40 | 0.28 | 0.17 | 0.10 | 0.02 | 0.02 | 0.01 |
| Cumulative Proportion | 0.40 | 0.68 | 0.86 | 0.95 | 0.97 | 0.99 | 1.00 |

**References**

Asheim J, Vike-Jonas K, Gonzalez SV, Lierhagen S, Venkatraman V, Veivåg I-LS, Snilsberg B, Flaten TP, Asimakopoulos AG. 2019. Benzotriazoles, benzothiazoles and trace elements in an urban road setting in trondheim, norway: Re-visiting the chemical markers of traffic pollution. Science of The Total Environment. 649:703-711.

Avagyan R, Sadiktsis I, Bergvall C, Westerholm R. 2014. Tire tread wear particles in ambient air—a previously unknown source of human exposure to the biocide 2-mercaptobenzothiazole. Environmental science and pollution research. 21(19):11580-11586.

Capolupo M, Sørensen L, Jayasena KDR, Booth AM, Fabbri E. 2020. Chemical composition and ecotoxicity of plastic and car tire rubber leachates to aquatic organisms. Water research. 169:115270.

Halsband C, Sørensen L, Booth AM, Herzke D. 2020. Car tire crumb rubber: Does leaching produce a toxic chemical cocktail in coastal marine systems? Frontiers in Environmental Science. 8:125.

Johnsen JP, Bye NH. 2019. Assessment of tire wear emission in a road tunnel, using benzothiazoles as tracer in tunnel wash water. Norwegian University of Life Sciences, Ås.

O’Brien AM, Lins TF, Yang Y, Frederickson ME, Sinton D, Rochman CM. 2021. A common contaminant shifts impacts of climate change on a plant-microbe mutualism: Effects of temperature, co2 and leachate from tire wear particles. bioRxiv.

Peter KT, Hou F, Tian Z, Wu C, Goehring M, Liu F, Kolodziej EP. 2020. More than a first flush: Urban creek storm hydrographs demonstrate broad contaminant pollutographs. Environmental Science & Technology. 54(10):6152-6165.

Unice KM, Bare JL, Kreider ML, Panko JM. 2015. Experimental methodology for assessing the environmental fate of organic chemicals in polymer matrices using column leaching studies and oecd 308 water/sediment systems: Application to tire and road wear particles. Science of The Total Environment. 533:476-487.

Zhang J, Zhang X, Wu L, Wang T, Zhao J, Zhang Y, Men Z, Mao H. 2018. Occurrence of benzothiazole and its derivates in tire wear, road dust, and roadside soil. Chemosphere. 201:310-317.
